# Simple Guanosine—Amino Acid Hybrids as Low Molecular Weight Hydrogelators

**DOI:** 10.1002/chem.70922

**Published:** 2026-04-03

**Authors:** Silvia Pieraccini, Samuele Ruffoli, Martina Occhi, Demetra Giuri, Devis Montroni, Claudia Tomasini, Stefano Masiero

**Affiliations:** ^1^ Dipartimento di Chimica “Giacomo Ciamician” Alma Mater Studiorum, Università Di Bologna Bologna Italy

**Keywords:** amino acids, gels, G‐quartet, guanosine, self‐assembly

## Abstract

While amino acids and nucleosides are the building blocks of molecular biology, very little is known about the behavior of simple hybrids consisting of a combination of these units. Here, we report the synthesis and self‐assembly of derivatives obtained by covalently linking guanosine (G) and amino acids (Gly and Ala). When triggered by a base, these hybrids behave as single‐component, low‐molecular‐weight hydrogelators through K^+^‐mediated formation of G‐quadruplexes, which are the supramolecular structural units responsible for physical gelation. The hydrogels were characterized by rheological measurements, circular dichroism (CD), and scanning electron microscopy (SEM). These transparent gels exhibit remarkable long‐term stability (months). Despite minimal differences in molecular structure, the conjugates form hydrogels with markedly distinct rheological and spectroscopic properties, enabling fine modulation of gel behavior. Overall, this work introduces a new class of guanosine‐based amino acid hydrogelators with potential applications spanning biomedicine and catalysis.

## Graphical abstract

Unexplored guanosine‐amino acid derivatives act as low molecular weight hydrogelators. Gelation is triggered by K^+^ ions and pH changes and relies on G‐quadruplex formation. Small changes in the amino acid structure have a strong impact on the mechanical properties of the gels.

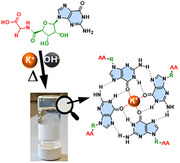

## 1 Introduction

**SCHEME 1:**
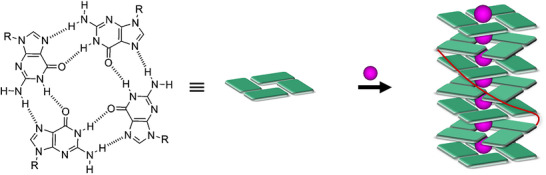
The tetrameric G4 structure (left), and a cartoon showing the cation (purple) mediated piling‐up of G4s to form a columnar G‐quadruplex (right).

Supramolecular hydrogels formed by low‐molecular‐weight gelators (LMWGs) have garnered great interest due to their dynamic nature and potential applications in biomedicine and materials science [1]. LMWGs self‐assemble through hierarchical noncovalent interactions, leading to nanostructured 3D networks capable of absorbing and retaining large amounts of water (often ≥ 98%–99% w/w). The resulting gel phase typically exhibits reversibility and self‐healing behavior [2]. Moreover, the physicochemical properties of these gels can often be tuned in response to external stimuli such as changes in pH, temperature, ionic strength, or component concentration, making these materials suitable for smart applications [3]. LMWGs are largely derived from natural products such as fatty acids, sugars, amino acids, peptides, and nucleosides [4, 5, 6, 7]. Among these, guanosine (G) and its derivatives have attracted growing attention because of their self‐assembly ability [8]. The guanine moiety possesses a unique sequence of hydrogen‐bond donors and acceptors, and can self‐associate into a variety of supramolecular arrangements (ribbons, sheets, helices, macrocycles) [9, 10]. Most G‐based hydrogels rely on the formation of the planar, tetrameric G‐quartet unit (G4), stabilized by the non‐canonical Hoogsteen‐type hydrogen bonding (Scheme 1) [9]. Piling of G4s gives rise to elongated, flexible columnar aggregates, the G‐quadruplexes (GQs), where adjacent aromatic planes undergo π–π stacking. A key stabilizing role is played by both pH and metal cations, typically Na^+^ or K^+^, which are coordinated within the central cavity to carbonyl oxygens.

Lateral column‐to‐column interactions and physical cross‐linking generate the supramolecular 3D network that drives the sol‐to‐gel transition [11]. The GQ architecture was first described by Gellert [12] based on fiber diffraction studies of GMP hydrogels. Since then, the GQ structure has been extensively studied and its biological significance widely recognized [13], motivating the development of GQ‐based soft materials. In recent years, hydrogels based on the GQ motif have emerged as highly versatile biomaterials, with applications in drug delivery, tissue engineering, and related biomedical technologies [14, 15, 16, 17, 18]. For the parent nucleoside G, hydrogelation is hindered by its low solubility and its strong tendency to crystallize [19, 20]. To overcome these limitations, a variety of modified guanosines have been developed that preserve G‐quadruplex formation while enhancing gel stability. In particular, binary mixtures of G with structurally related analogues, such as 5′‐GMP [11, 21], 2′‐deoxyguanosine [22], and other derivatized guanosines [23, 24, 25], have yielded long‐lived hydrogels. High‐performance materials have been obtained from 2′,3′‐G‐borate diesters in the presence of suitable cations, where the borate anion enhances aqueous solubility and covalent G–G linkage promotes network formation [14]. The co‐incorporation of different G analogues is thought to introduce structural disorder [26], which suppresses spontaneous crystallization [23, 25], while an appropriate balance between hydrophilicity and hydrophobicity has also proven critical for effective gelation [11, 21].

Combining the above factors in a single compound requires a careful design: this is probably the reason why only a limited number of modified guanosines have been shown to efficiently gel water in the absence of co‐formulants, highlighting the interest in new single‐component GQ hydrogelators. Among these, functional hydrogelators have been reported by Lehn [27, 28, 29] and Davis [18, 30]. While the structural scope and functional tunability of single‐component GQ hydrogels are still limited, these latter advances open the way to a large range of possibilities.

Amino acids and nucleosides represent the fundamental building blocks of life, and the study of their mutual covalent or noncovalent interactions remains an especially relevant topic, due to possible implications in prebiotic chemistry and the origin of protein biosynthesis. In ribosome‐catalyzed peptide synthesis, transfer RNA is first covalently aminocylated and then undergoes the polymerization process [31]. In spite of the relevance of these intermediates, research efforts were more focused on more complex nucleopeptides [32, 33], while very little has been reported in the literature concerning simple covalent hybrids between amino acids and nucleosides [34, 35, 36].

**FIGURE 1:**
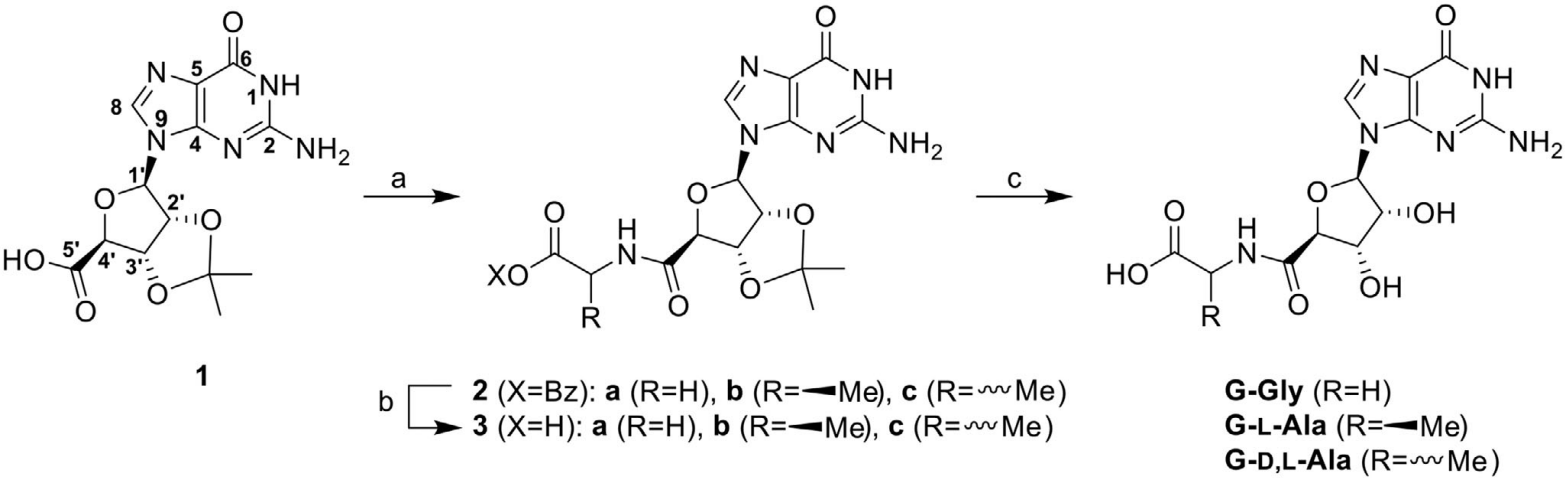
Synthesis of the guanosine/amino acid hybrids and their chemical structures. Conditions: (a) amino acid benzyl ester hydrochloride, TBTU, Et_3_N, DMF, r.t., 3.5–5 h; (b) H_2_, Pd(OH)_2_ 20% on carbon, MeOH, r.t., 3–4 h; (c) H_2_O/HCOOH 1:1, 70°C, 2.5–3.5 h. Atom numbering shown in Structure **1** follows the IUPAC numbering of guanosine, which is used throughout the text.

Herein, we report on three new, unexplored guanosine derivatives, covalently bound at the primary 5’‐position with amino acids: glycine, L‐alanine, and D,L‐alanine (Figure 1). We explored the ability of these hybrids to gel water in the presence of K^+^ ions, thus evaluating the effect of the different aminoacidic residues. The resulting hydrogels were characterized by rheological and spectroscopic analyses, as well as by SEM imaging.

## 2 Results and Discussion

### 2.1 Synthesis and Characterization of G‐AA Derivatives

The synthesis of the three hybrids (Figure 1) was carried out starting from commercial guanosine and amino acid benzyl esters by adapting common procedures. Guanosine was first protected as 2′‐3′‐isopropylidene acetal, then oxidized at the primary alcoholic function to afford **1**, following published procedures [37, 38]. The acid **1** thus obtained was condensed with the amino acid benzyl ester hydrochloride of choice in the presence of 2‐(1H‐Benzotriazole‐1‐yl)‐1,1,3,3‐tetramethylaminium tetrafluoroborate (TBTU) and triethylamine to give the protected amides **2a–c**. Subsequent hydrogenolysis with Pd/H_2_ of the benzyl esters and acid‐catalysed hydrolysis of the acetals gave the three target products. Synthetic details and analytical characterization of the compounds are reported in section 1 and related subsections of Supporting Information.

### 2.2 Formation of Supramolecular Gels

**FIGURE 2:**
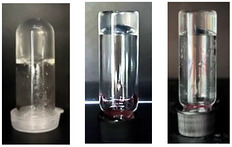
Photos of results obtained after the process of gel formation described above. From left to right: **G‐Gly**, **G‐L‐Ala**, **G‐D,L‐Ala**.

To induce the supramolecular interactions responsible for the formation of a physical gel, it is often necessary to add a trigger. In the case of this group of molecules, two possible triggers are available. Indeed, the guanosine unit is sensitive to the addition of monovalent cations that induce the formation of quadruplexes. Moreover, pH variation allows protonation/deprotonation of the carboxyl unit to achieve the optimal balance between hydrophilic and hydrophobic components. In this work, we took advantage of the modulation of both triggers by using solutions of both KOH and KCl. The choice of K^+^ ions was due to their known ability to template GQ self‐assembly, while the addition of OH^−^ ions favored dissolution in water of the compounds through partial deprotonation of the carboxyl group. After several attempts varying the equivalents of the two triggers to find the best conditions for gel formation (results not shown), we selected the following: addition of 0.75 equivalents of KOH and 7.8 equivalents of KCl to the gelator aqueous 1.5% w/V suspension. The mixtures were sonicated or magnetically stirred for 5 min, then heated to 90°C for 5 min to dissolve the organic molecules. The solutions were then allowed to stand quiescently for 16 h at r.t. The three samples obtained from **G‐Gly**, **G‐L‐Ala**, **G‐D,L‐Ala** readily formed gels with a highly transparent appearance (Figure 2). In all cases, the final pH of the gels was 4.0‐4.2. These clear, self‐standing gels remained stable over long periods (weeks), not showing any change in their transparent appearance nor water loss (Figure S1). Upon dilution of these gels with progressive additions of 0.35 M KCl, self‐standing gels were obtained at gelator concentrations as low as 40 mM after a heating–cooling cycle. Control experiments carried out with 0.75 equivalents of KOH but with no KCl added led to transparent soft gels, which gave a positive inversion test, but were unable to support the weight of the stirring bar, eventually contained in the vial. On the other hand, heating the hybrid in the presence of KCl but without the proper amount of KOH, followed by cooling to r.t., led to the formation of a precipitate, highlighting the crucial role of deprotonation in gel formation. It is worth pointing out that, with respect to GQ formation, 0.25 equivalents of KOH are sufficient to provide the required amount of K^+^ for a 4:1 G/K^+^ stoichiometry. Stable gels were also obtained when 0.75 equivalents of KOH but only 1 equivalent of KCl were used: this means gelation can occur at intracellular K^+^ concentrations and potassium in excess of its stoichiometric requirement contributes to strengthening the gel network.

A preliminary evaluation performed through dilution experiments highlighted the superior gelation ability of the **G‐Gly** derivative. The three 1.5% hydrogels were diluted to 1% and 0.5% w/v, and were classified using the tube inversion test after being stored at either r.t. or at 5°C for 1 h. As summarized in Table 1, **G‐Gly** still forms a self‐supporting gel at 1% w/v, while at 0.5% w/v it shows thermodissociative behavior: [12] indeed, it is a viscous solution at r.t., whereas it gels at 5°C. **G‐L‐Ala** and **G‐D,L‐Ala** behave similarly upon dilution. At 1% w/v, they both are viscous solutions at r.t., whereas they form gels at 5°C. Different from **G‐Gly**, at 0.5% w/v, no gelation is observed: both systems appear as clear solutions at r.t. and become viscous at 5°C.

**TABLE 1:** Classification of **G‐Gly**, **G‐L‐Ala,** and **G‐D,L‐Ala** samples at different concentrations and temperatures, as clear solutions (open circles), viscous solutions (open triangles), and gels (solid squares). The 1% and 0.5% w/v samples were obtained by diluting the 1.5% w/v hydrogels with the KCl solution. The graphical symbols were adopted from ref [21].

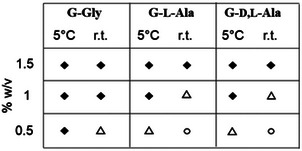

### 2.3 Rheological Studies

**FIGURE 3:**
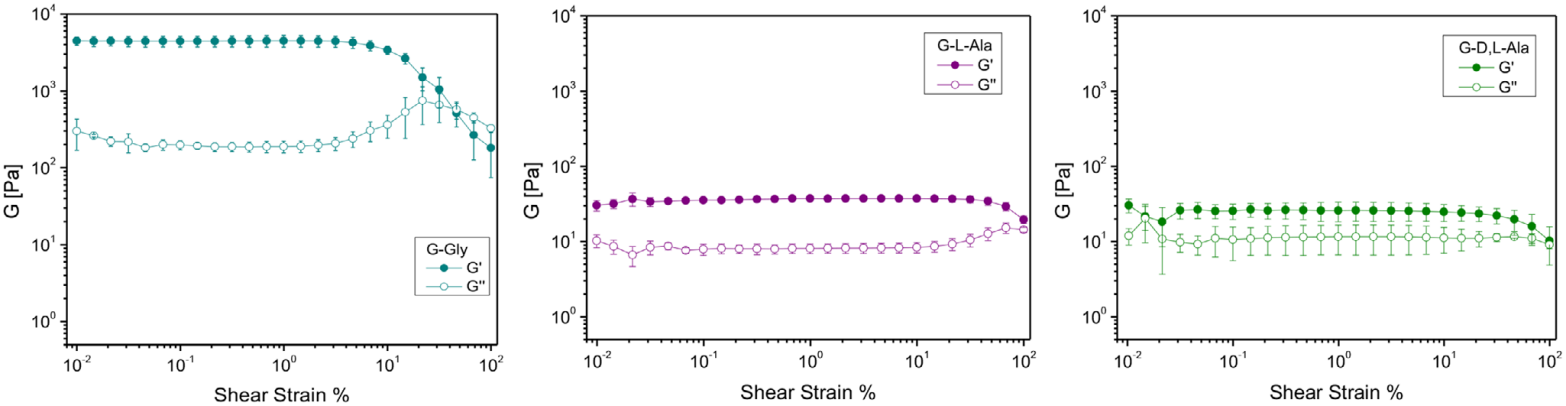
Amplitude sweep tests of the hydrogels at 1.5% w/V concentration. From left to right: **G‐Gly, G‐L‐Ala, G‐D,L‐Ala**.

Rheological measurements allowed us to gain information on the different viscoelastic properties of the gels. The amplitude (Figure 3) and frequency (Figure S2) sweep tests confirmed the gel‐like behavior of the three samples, which have G′ values always higher than the G″ and a large linear viscoelastic region (LVE region), the plateau range where both G′ and G″ are independent of the strain applied and result linear. The frequency sweep confirms this outcome, showing that below the critical strain, at a constant γ of 0.01% (within the LVE region), the elastic modulus G′ is nearly independent of frequency, as would be expected from a structured solid‐like material. The stiffness and elasticity of the three samples are very different, as reported in Table 2. The G′ modulus of the **G‐Gly** gel exceeds that of the Ala analogues by one order of magnitude. In contrast, the two Ala derivatives are much more elastic, having a crossover point (G′ = G″) close to γ = 100%. The gels obtained from the diastereoisomeric mixture **G‐D,L‐Ala** show a similar stiffness compared to the **G‐L‐Ala** analogue, but decreased reproducibility (increased standard deviation). This result can be easily explained, considering that the diasteroisomeric mixture suffers from some variation on the ratio between the two isomers, due to the purification step that may enrich the amount of one diastereoisomer compared to the other.

**TABLE 2:** Summary of G′ and G″ (γ = 0.046%) values of the amplitude sweep tests performed in triplicate, reported as mean ± standard deviation.

Gelator	G' (Pa)	G" (Pa)
G‐Gly	4407.2 ± 702.4	182.8 ± 20.8
G‐L‐Ala	34.5 ± 2.0	8.7 ± 0.9
G‐D,L‐Ala	26.7 ± 6.4	9.3 ± 2.6

The three samples were further characterized to assess their ability to recover their original properties after heating (thermoreversibility) or shear stress (thixotropy) by evaluating the time‐dependent recovery of their initial properties.

**FIGURE 4:**
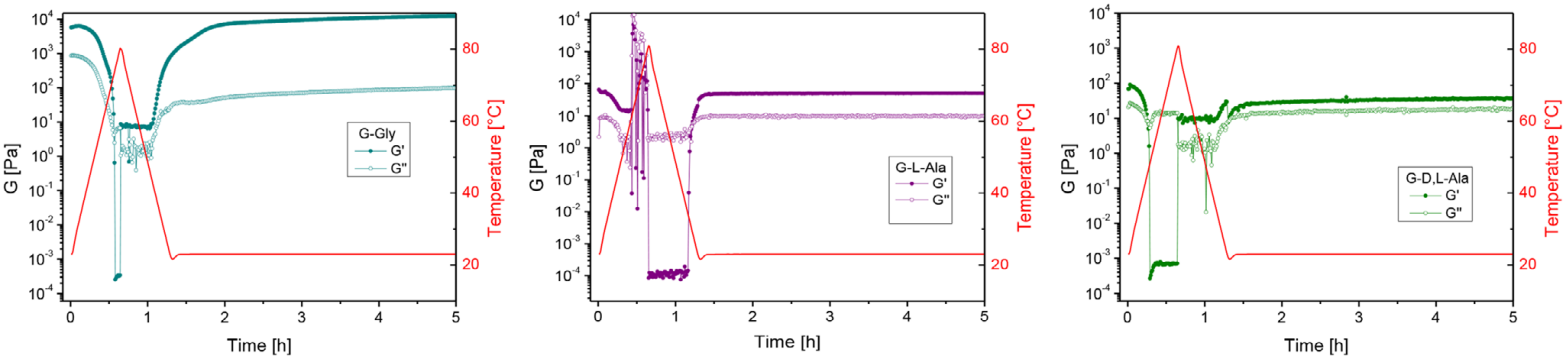
Time sweep test of the hydrogels at 1.5% w/V. From left to right: **G‐Gly**, **G‐L‐Ala**, **G‐D,L‐Ala**.

In Figure 4, we show the results of the variation of the G′ and G″ values as a function of the temperature in a range between 23°C and 80°C, using a constant angular frequency of 10 rad/s and γ = 1%. After a peak of temperature of 80°C, the same temperature range was set to cool the sample to 23°C. To assess the ability of the materials to recover their properties with time, the samples were analyzed for a total time of 3.5 h after reaching 23°C. All the samples recover the starting properties immediately after cooling down and maintain them.

**FIGURE 5:**
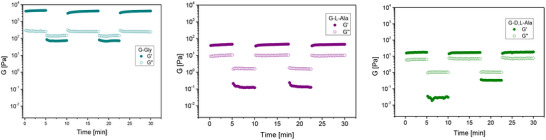
Thixotropy sweep test of the hydrogels at 1.5% w/V. From left to right: **G‐Gly**, **G‐L‐Ala**, **G‐D,L‐Ala**.

The thixotropic behavior of the hydrogels was assessed with strain‐recovery experiments, subjecting the samples to consecutive deformation and recovery steps (Figure 5). The first step (rest conditions) was performed at a constant strain γ = 0.5% (within the LVE region) and at a fixed frequency of ω = 10 rad s^−1^ for a period of 300 s. The deformation step was performed applying a constant strain of γ = 800% (above the LVE region) for a period of 300 s. During this step of high shear, the gel network breaks down (G″ > G′). The recovery step was performed with the same conditions as the first step for a period of 450 s. Deformation and recovery steps were repeated two times. Also in this case, the material's properties were fully recovered, confirming the exceptional viscoelastic properties of all these materials.

### 2.4 SEM Analysis

**FIGURE 6:**
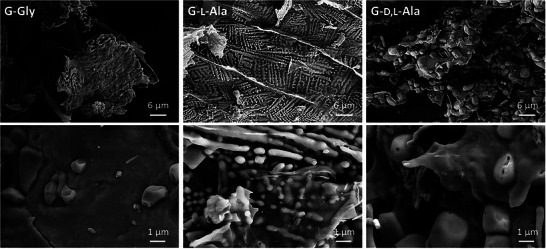
SEM images of the three unwashed freeze‐dried hydrogels. For each sample, images at lower (top) and higher (bottom) magnifications are shown.

**FIGURE 7:**
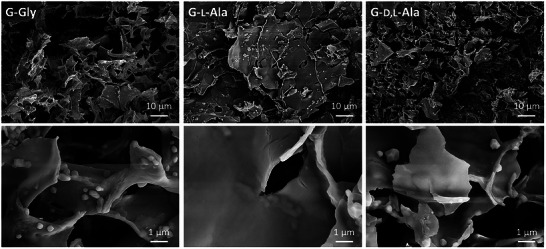
SEM images of the three freeze‐dried hydrogels washed with water. For each sample, images at lower (top) and higher (bottom) magnifications are reported.

To gain insight about the morphology of the gels, the samples were freeze‐dried prior to SEM (scanning electron microscopy) analysis (Figure 6). Phases with crystalline morphologies are clearly identifiable, likely arising from inorganic crystals formed by the deposition of KOH and KCl, the latter being present in large amounts relative to the gelator during lyophilization. These crystals extensively cover the sample and, particularly in the **G‐L‐Ala** sample, appear to nucleate within the organic material, potentially leading to aberrations or structural modifications of the sample. Although the morphology of the organic phase remains partially visible, the aerogels were washed with water (see Section S4) to remove residual salts and improve observation of the organic matrix. (Figure 7).

SEM observations of the washed freeze‐dried hydrogels revealed a thin sheet‐like morphology in all three samples. Although the sheet thickness was on the order of several hundred nanometres for all samples, **G‐Gly** and **G‐D,L‐Ala** exhibited comparable sheet extensions, whereas **G‐L‐Ala** showed a noticeably larger one. From a morphological standpoint, **G‐Gly** displayed more highly branched sheets with a greater degree of irregularity compared to the other two samples. This irregularity appears to lead to a higher interconnectivity between sheets, which may be contributing to the higher mechanical resistance of the corresponding hydrogels. At higher magnification, no distinct nanometric features were observed.

### 2.5 ECD/UV Studies

**FIGURE 8:**
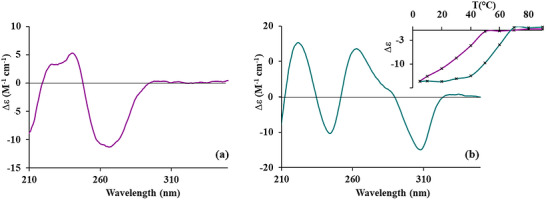
ECD spectra of 1.5% w/v hydrogels of **G‐L‐Ala (a)** and **G‐Gly (b)** recorded at 20°C in a 0.001 cm cell. In the inset, Δε values at 263 nm for **G‐L‐Ala** and 308 nm for **G‐Gly** are plotted against temperature.

Electronic Circular Dichroism (ECD) spectroscopy is highly sensitive to the structural and conformational features of chiral supramolecular assemblies, and has become a primary tool for studying both covalent and noncovalent GQ architectures [39, 40, 41]. ECD spectra recorded on 1.5% w/v hydrogels of **G‐L‐Ala** and **G‐Gly** at 20 °C are reported in Figure 8.

They both exhibit exciton signals within the guanine absorption region, diagnostic of chirally stacked G‐quartets. The spectrum of **G‐L‐Ala** displays a nonconservative negative exciton couplet centered at 248 nm, with a negative band at 263 nm and a less intense positive band at 240 nm. This signature is generally ascribed to left‐handed quadruplexes [42, 43]. In contrast, the **G‐Gly** hydrogel shows a strikingly different profile. A nearly symmetric, positive exciton couplet is centered at ca. 250 nm (cross‐over 252 nm), with a positive maximum at 262 nm and a negative minimum at 244 nm. Moreover, an additional well‐defined negative Cotton effect appears at around 308 nm. Both curves show a positive‐to‐negative trend below 235 nm. Overlaying the spectra (Figure S3a) shows that the **G‐L‐Ala** and **G‐Gly** profiles are almost mirror images between 290 and 235 nm, while they differ markedly above 290 nm. Notably, a similar spectral relationship has been described for quadruplexes sharing the same helicity but differing in stacking polarity (see Figure S4 and caption therein) [40, 41]. Specifically, two oppositely signed bands at ca. 260 and 240 nm have been ascribed to homopolar G4 stacking. Conversely, three bands at ca. 290, 260, and 240 nm, showing alternate signs, have been correlated with heteropolar stacking. On this basis, in the **G‐L‐Ala** hydrogel, the G4 units likely stack with a homopolar (head‐to‐tail) orientation, whereas in **G‐Gly**, a heteropolar stacking can be tentatively proposed, with head‐to‐head and tail‐to‐tail connections alternating along the columns [44]. The ECD spectrum of the 1.5 % w/v hydrogel of **G‐D,L‐Ala** at 20°C, compared to that of **G‐L‐Ala**, is shown in Figure S3b. The two traces display similar band shapes, consistent with hydrogels based on GQs with the same helicity and stacking polarity. The lower intensity of the signal detected for the diastereoisomeric mixture is consistent with a reduced degree of supramolecular order. At the macroscopic level, this translates into the more fluid‐like character of the gel arising from the diastereomeric mixture, as suggested by the smaller separation between G′ and G″ values observed in the rheological experiments.

To explore the self‐assembly process underlying gel formation, variable‐temperature ECD/UV measurements were performed upon cooling **G‐L‐Ala** and **G‐Gly** hydrogels at 1.5% w/v (Figure S5). UV spectra of the two dissolved samples at 90°C are nearly superimposable (Figure S6), and display the two characteristic π‐π* electronic transitions of guanine at around 278 and 253 nm, roughly short and long axis polarized, respectively [45]. As the temperature decreases and the hydrogel forms, a significant hypochromic effect develops at the absorption maximum (*ca*. 35% decrease from 90 to 5°C), typically associated with guanosine gel formation [46]. At 5°C, some features differentiate the spectra, presumably related to the different stacking polarities. For **G‐Gly** the maximum is red‐shifted by 4 nm compared to **G‐L‐Ala**, and hyperchromism above 300 nm, indicative of G‐quartet formation, is more pronounced [47]. This absorption likely accounts for the optical activity noted at this wavelength. Considering the ECD traces (Figure S5), at 90°C the signal is negligible due to dissociation of the G4‐based aggregates, while the distinctive bands gradually intensify on cooling. ECD intensities plotted against temperature at 263 nm for **G‐L‐Ala** and 308 nm for **G‐Gly** are shown in the inset of Figure 8. For **G‐Gly**, ellipticity remains close to zero down to 70°C, where an abrupt drop marks the onset of the GQ refolding (*T*_on_). Differently, for **G‐L‐Ala**, *T*_on_ occurs at 50°C, likely reflecting weaker stabilizing interactions within the GQ network. Moreover, for **G‐Gly,** the curve displays a sigmoidal shape with a steep slope, indicative of a highly cooperative self‐assembly, whereas the **G‐L‐Ala** profile evolves more gradually. Overall, at 40°C, **G‐Gly** has reached 90% of the final ECD intensity, while **G‐L‐Ala** has only 30%. These data point to a less cooperative and slower GQ assembly for **G‐L‐Ala** relative to **G‐Gly**, suggesting that differences in stacking polarity may be associated with intrinsically different self‐assembly pathways and GQ‐formation kinetics. For both samples, reversibility of GQ self‐assembly was verified by subsequent melting of the gel at 90°C followed by cooling to 20°C, yielding essentially superimposable CD spectra (see Figure S7).

## 3 Conclusions

In this paper, we described the synthesis and the behavior of unexplored, simple guanosine‐amino acid hybrids. When triggered with base and K^+^ ions, the compounds act as single‐component hydrogelators at concentrations down to 1% w/V (for **G‐Gly**). The resulting transparent gels are stable over a long period of time and exhibit full thermoreversibility and thixotropy. ECD studies show that G‐quadruplexes are the supramolecular architectures responsible for the formation of these physical gels. Small structural differences in the amino acid residue have a strong impact on the mechanical properties of the corresponding hydrogels, and this appears to be linked to the different G‐quadruplex topologies originating from the different hybrids. These results reveal that covalent conjugation of amino acids to guanosine provides a versatile strategy to tune hydrogel properties. More generally, they suggest a new family of guanosine‐based hydrogelators, in which guanosines suitably derivatized with unprotected amino acid residues self‐assemble into robust and tunable G‐quadruplex architectures. These organized materials expose amino acid residues, enabling modulation of their structural, mechanical, and functional properties, with potential applications ranging from biomaterials to catalysis.

## Conflicts of Interest

The authors declare no conflict of interest.

## Supporting information




**Supporting File**: chem70922‐sup‐0001‐SuppMat.pdf.

## Data Availability

Full experimental procedures, synthesis and characterization of the compounds, gelation protocol, additional rheological data, UV and CD spectroscopic analyses are available in the supplementary material of this article. The authors have cited additional references within the Supporting Information [48].
