# Supplementary material for: Simple Guanosine—Amino Acid Hybrids as Low Molecular Weight Hydrogelators
Source: Chemistry. 2026 Apr 3;32(23):e70922. doi: 10.1002/chem.70922 (PMC13282916; doi:10.1002/chem.70922)
Supplement: Supplementary file 1 — Supporting File: chem70922‐sup‐0001‐SuppMat.pdf. [file CHEM-32-e70922-s001.pdf]

Supplemental information for:

Simple guanosine – amino acid hybrids as low molecular weight hydrogelators

Silvia Pieraccini, Samuele Ruffoli, Martina Occhi, Demetra Giuri, Devis Montroni, Claudia Tomasini, Stefano Masiero*

Dipartimento di Chimica "Giacomo Ciamician" - Alma Mater Studiorum - Università di Bologna
Plesso "Navile" – UE4, Via Piero Gobetti, 85 - 40129 – Bologna (Italy)

stefano.masiero@unibo.it

Index

1. **Synthetic procedures** 2

1a. General procedure for the synthesis of **2** (**Gace_AA_OBz**) 3
2a Gace_Gly_OBz 3
2b (from L-alanine) **Gace_L_Ala_OBz** 6
2c (from D,L-alanine) **Gace_D,L_Ala_OBz** 8
1b. General procedure for the synthesis of **3** (**Gace_AA**) 11
3a Gace_Gly 11
3b (from -L-alanine) **Gace_L_Ala** 13
3c (from D,L-alanine) **Gace_D,L_Ala** 16
1c. General procedure for the synthesis of **G_AA** 18
G-Gly 18
G-L-Ala 22
G-D,L-Ala 25

2. **General procedure for gels preparation** 28

3. **General procedure for the rheology analysis** 28

Figure S1. G-Gly gels at 4% and 1.3% w/v 29
Figure S2: Thixotropy sweep test of gels of **G-L-Ala, G-Gly**, **G-D,L-Ala** 30

4. **Procedures for SEM analysis** 32

5. **General procedures for ECD/UV analysis** 32

Figure S3. ECD spectra of gels of **G-L-Ala, G-Gly**, **G-D,L-Ala** 31
Figure S4. G-quartets and stacking polarity 32
Figure S5. (*VT*)-ECD/UV spectra of **G-L-Ala** and **G-Gly** hydrogels 33
Figure S6. UV spectra of **G-L-Ala** and **G-Gly** gels at 5°C and 90°C 33
Figure S7. ECD spectra of **G-L-Ala** and **G-Gly** gels at 20°C and 90°C 34

1. Synthetic procedures

General

All reactions were carried out under magnetic stirring. Reactions requiring anhydrous conditions were carried out in oven-dried glassware under a dry argon atmosphere. For TLC analyses, Baker IB2-F silica gel plates were used. Column chromatography was performed on Aldrich silica gel 230-400 mesh. Reagents and solvents, including dry solvents, were purchased from Merck or TCI. Triethylamine was dried by distillation over CaH_2 and stored over molecular sieves under Ar. 2',3'-O-isopropylideneguanosine 5'-carboxylic acid **1** (scheme S1) was prepared from commercial guanosine according to literature procedures.[37,38]

NMR spectra were recorded with Varian Inova (300 or 600 MHz), Mercury (400 MHz) or Bruker Avance NEO (600 MHz) instruments. NMR spectra were referenced relative to residual solvent peaks. Signals were assigned on the basis of COSY, HSQC and HMBC experiments (s=singlet, bs=broad singlet d=doublet, t=triplet, q=quartet, p=quintet, dd= double doublet, m=multiplet). For numbering of guanosine's protons and carbons refer to compound **1** in Scheme S1.

Electrospray ionization (ESI) mass spectra were acquired on a Micromass ZQ-4000 instrument by direct infusion of a methanol solution. High resolution mass spectra MALDI/Q-TOF were recorded with a SYNAPT GS2 spectrometer.

Synthesis

The synthetic procedures employed to obtain the guanosine-amino acid hybrids are outlined in Scheme 1.

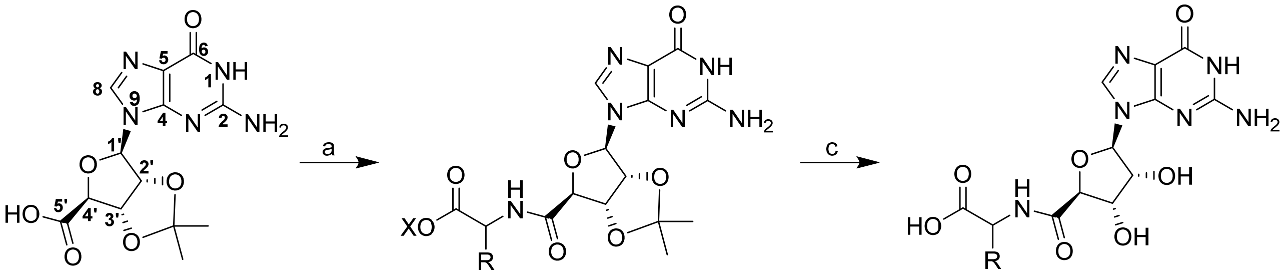

1

b **2** (X=Bz): **a** (R=H), **b** (R=◄Me), **c** (R=~Me)
3 (X=H): **a** (R=H), **b** (R=◄Me), **c** (R=~Me)

G-Gly (R=H)
G-L-Ala (R=◄Me)
G-D,L-Ala (R=~Me)

Scheme S1: synthesis of the guanosine/amino acid hybrids. Conditions: (a) amino acid benzyl ester hydrochloride, TBTU, Et_3N, DMF, r.t., 3.5-5h; (b) H_2, $Pd(OH)_2$ 20% on carbon, MeOH, r.t., 3-4h; (c) H_2O/HCOOH 1:1, 70°C, 2.5-3.5h.

1a - General procedure for the synthesis of **2** (**Gace_AA_OBz**):

Acid **1** (0.543 g, 1.60 mmol) was suspended in dry DMF (20 mL) with the aid of an ultrasonic bath. Et_3N (0.50 mL, 3.6 mmol) and TBTU (0.565 g, 1.76 mmol) were added and the resulting milky mixture was stirred for 40 min at r.t. The amino acid benzyl ester hydrochloride (1.80 mmol) was then added and stirring was continued at r.t. until ESI-MS analysis confirmed the disappearance of the guanosine starting material (3.5-5 hrs). The crude reaction mixture was then poured into water (40 mL) under vigorous stirring and the resulting precipitate was filtered. Usually, a second crop of product could be obtained by leaving the mother liquors in the fridge overnight. The combined solids were dried in vacuo and purified by column chromatography on silica gel.

2a (**Gace_Gly_OBz**)

White solid, 60% yield, CH_2Cl_2: MeOH 9:1, r.f. 0.37.

^{1}H NMR (400 MHz, DMSO-d_6) δ 10.64 (bs, 1H, guanine NH), 8.02 (t, *J* = 5.9 Hz, 1H, -CH_2-NH-CO-), 7.84 (s, 1H, H8), 7.46 – 7.24 (m, 5H, Ph), 6.37 (bs, 2H, NH_2), 6.12 (d, *J* = 2.0 Hz, 1H, H1'), 5.30 (dd, *J* = 6.2, 2.7 Hz, 1H, H3'), 5.21 (dd, *J* = 6.2, 2.0 Hz, 1H, H2'), 5.09 (s, 2H, Ph-CH_2), 4.56 (d, *J* = 2.7 Hz, 1H, H4'), 3.88 and 3.63 (dd, dd, *J* = 17.4, 6 Hz, 2H, CH_2-NH-CO), 1.53 (s, 3H, CH_3), 1.32 (s, 3H, CH_3).

^{13}C NMR (75 MHz, DMSO-d_6) δ 169.25 (5'CONH), 169.16 (COOBz), 156.70, 153.63, 150.68 (C4), 136.18 (C8), 135.78 (Ph), 128.41 (Ph), 128.08 (Ph), 127.95 (Ph), 116.71 (C5), 113.22 (CMe_2), 88.82 (C1'), 85.32 (C4'), 83.41 (C2'), 82.90 (C3'), 65.92 (Ph-CH_2), 40.35 (CH_2-NH-CO), 26.75 (CH_3), 25.10 (CH_3).

HRMS (MALDI/Q-TOF) m/z: $[M + H]^+$ calcd. for $C_{22}H_{25}N_6O_7$ 485.1785, found 485.1789.

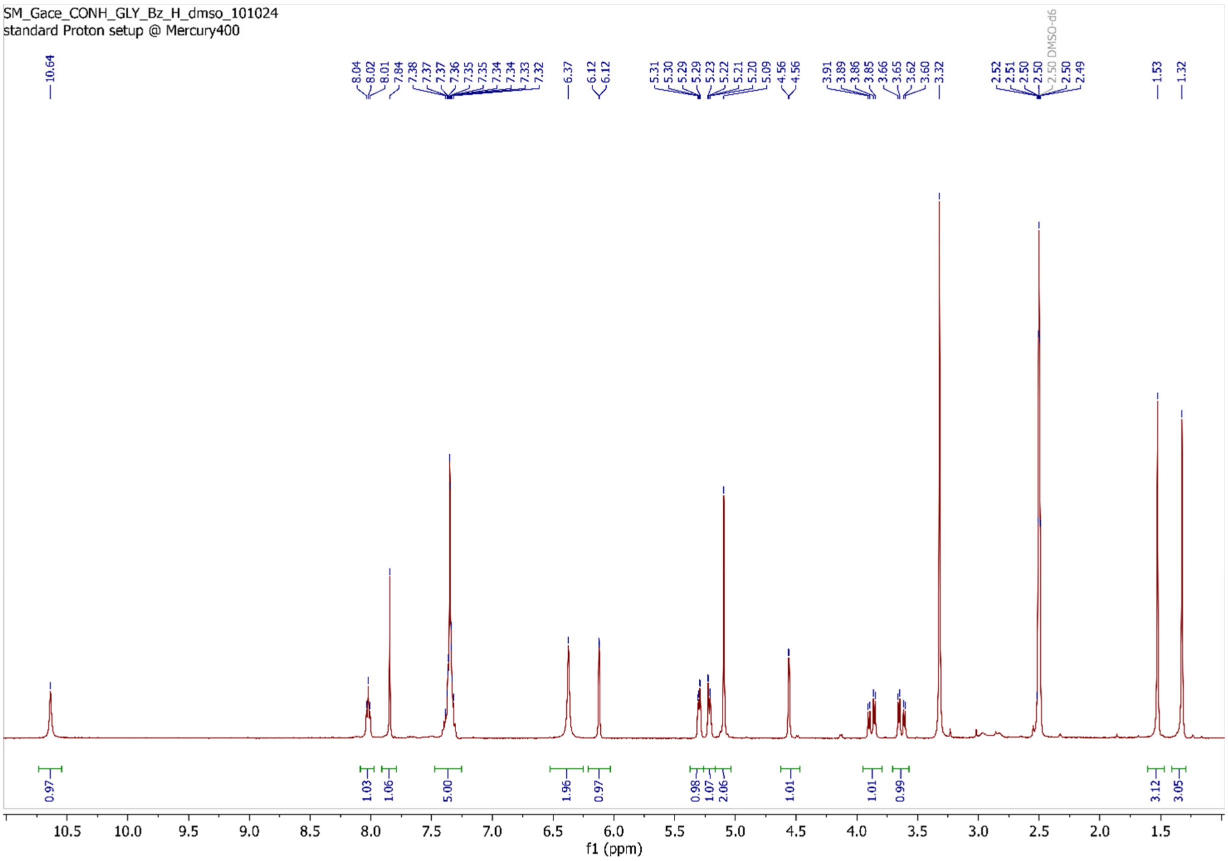

^{1}H-NMR spectrum of **2a** in DMSO-d_6

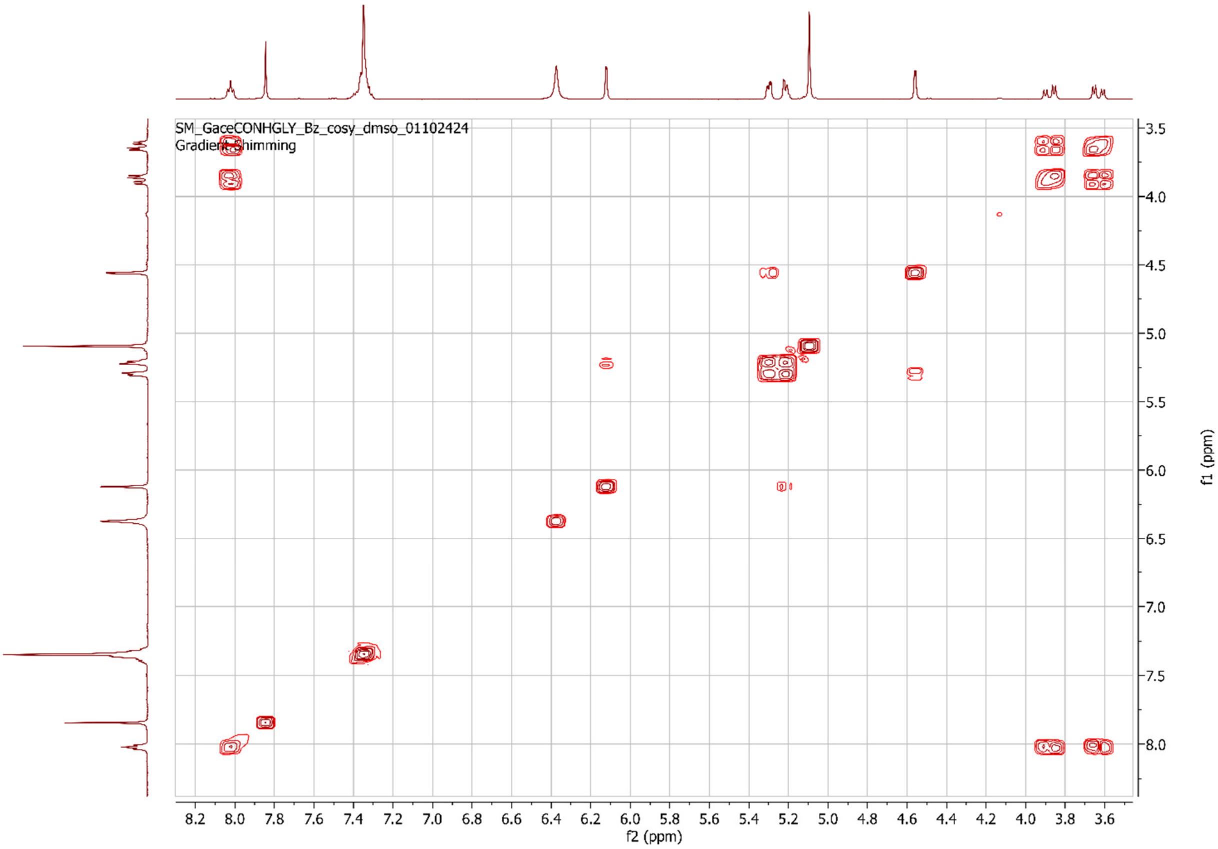

COSY spectrum of **2a** in DMSO-d_6

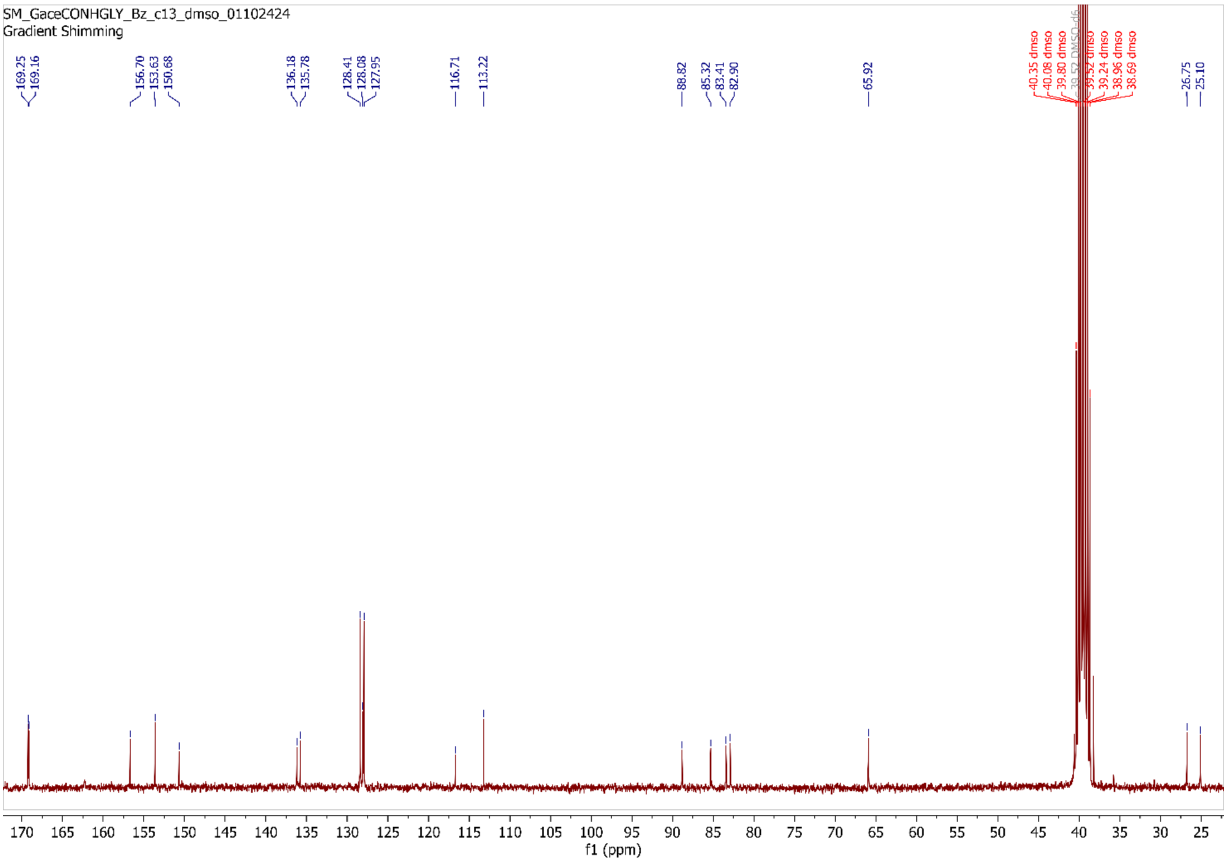

^{13}C-NMR spectrum of **2a** in DMSO-d_6

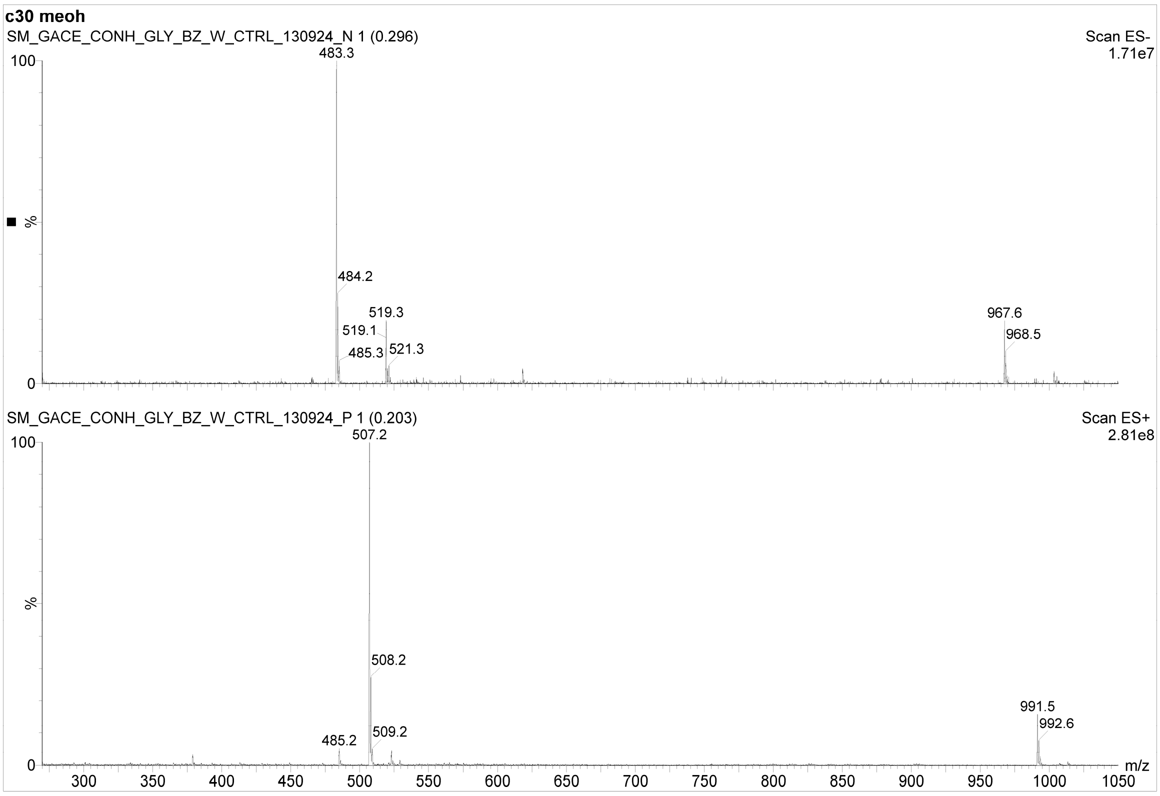

ESI-MS spectra of **2a**

2b (from L-alanine) (**Gace_L_Ala_OBz**)

White solid, 83% yield, CH_2Cl_2: MeOH 95:5, r.f. 0.28.

^{1}H NMR (600 MHz, DMSO-*d*$_6$) δ 10.64 (bs, 1H, guanine NH), 7.83 (s, 1H, H8), 7.75 (d, *J* = 7.3 Hz, 1H, -CH-NH-CO-), 7.38 – 7.29 (m, 5H, Ph), 6.41 (bs, 2H, NH_2), 6.17 (d, *J* = 1.4 Hz, 1H, H1'), 5.43 (dd, *J* = 6.1, 2.4 Hz, 1H, H3'), 5.27 (dd, *J* = 6.1, 1.4 Hz, 1H, H2'), 5.12 – 5.03 (m, *J* = 9.7 Hz, 2H, CH_2), 4.53 (d, *J* = 2.4 Hz, 1H, H4'), 4.20 (p, *J* = 7.3 Hz, 1H, NH-CH-CO), 1.51 (s, 3H, C^{IV}-CH_3), 1.33 (s, 3H, C^{IV}-CH_3), 1.00 (d, *J* = 7.3 Hz, 3H, CH-CH_3).

^{13}C NMR (75 MHz, DMSO-*d*$_6$) δ 171.77 (COOBz), 168.61 (5'CONH), 156.66, 153.43, 150.64 (C4), 136.59 (C8), 135.87 (Ph), 128.42 (Ph), 128.01 (Ph), 127.65 (Ph), 116.66 (C5), 112.78 (CMe_2), 89.09 (C1'), 85.98 (C4'), 83.54 (C2'), 83.21 (C3'), 65.96 (Ph-CH_2), 47.30 (CH-NH-CO), 26.64 (CH_3), 25.07 (CH_3), 16.11 (CH-CH_3).

HRMS (MALDI/Q-TOF) m/z: $[M + H]^+$ calcd. for $C_{23}H_{27}N_6O_7$ 499.1941, found 499.1945.

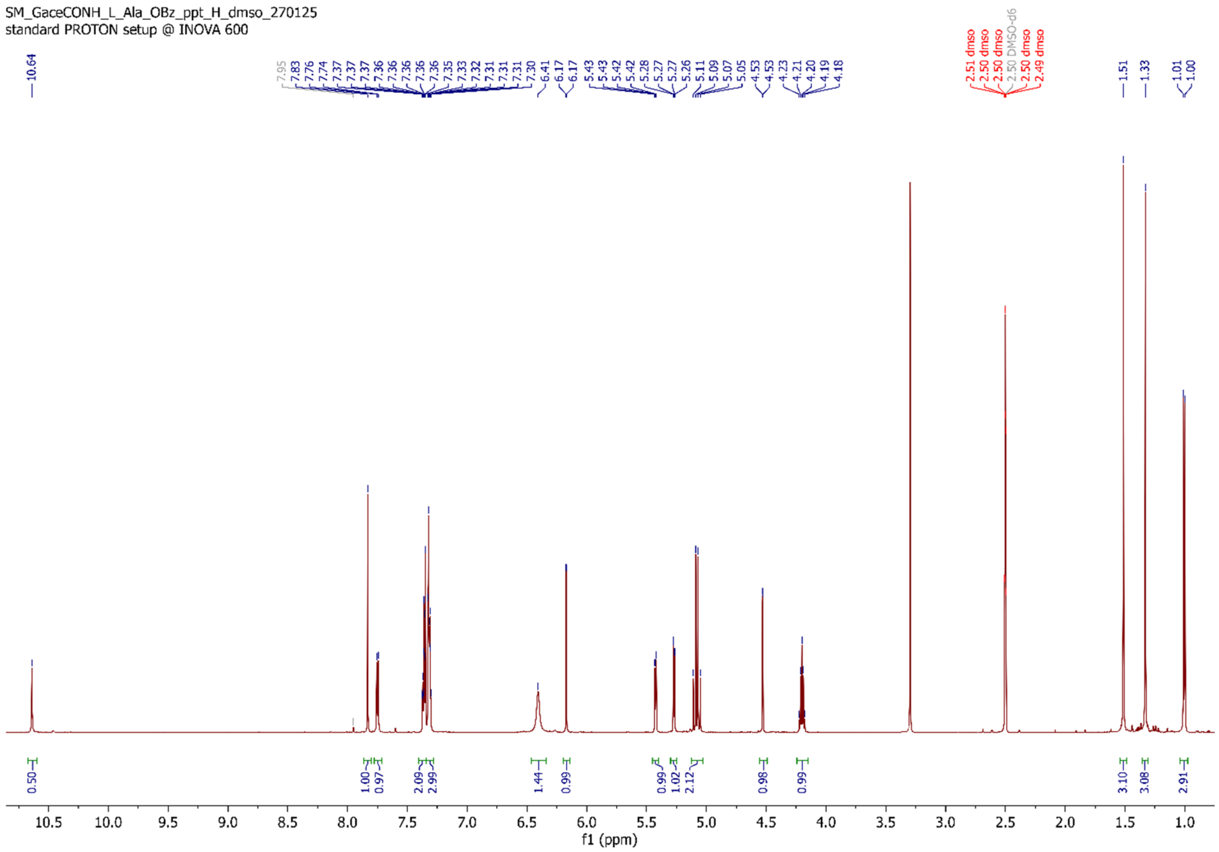

^{1}H-NMR spectrum of **2b** in DMSO-*d*$_6$

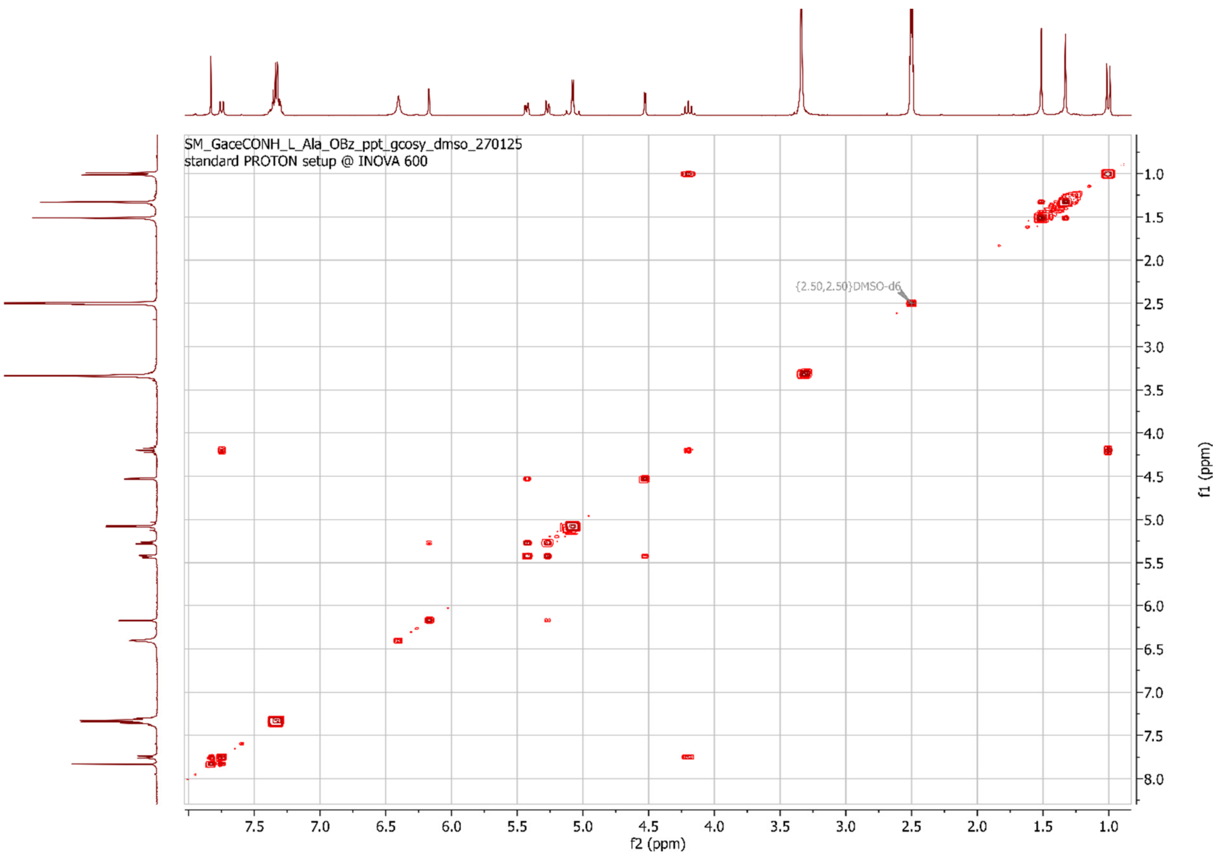

COSY spectrum of **2b** in DMSO-d_6

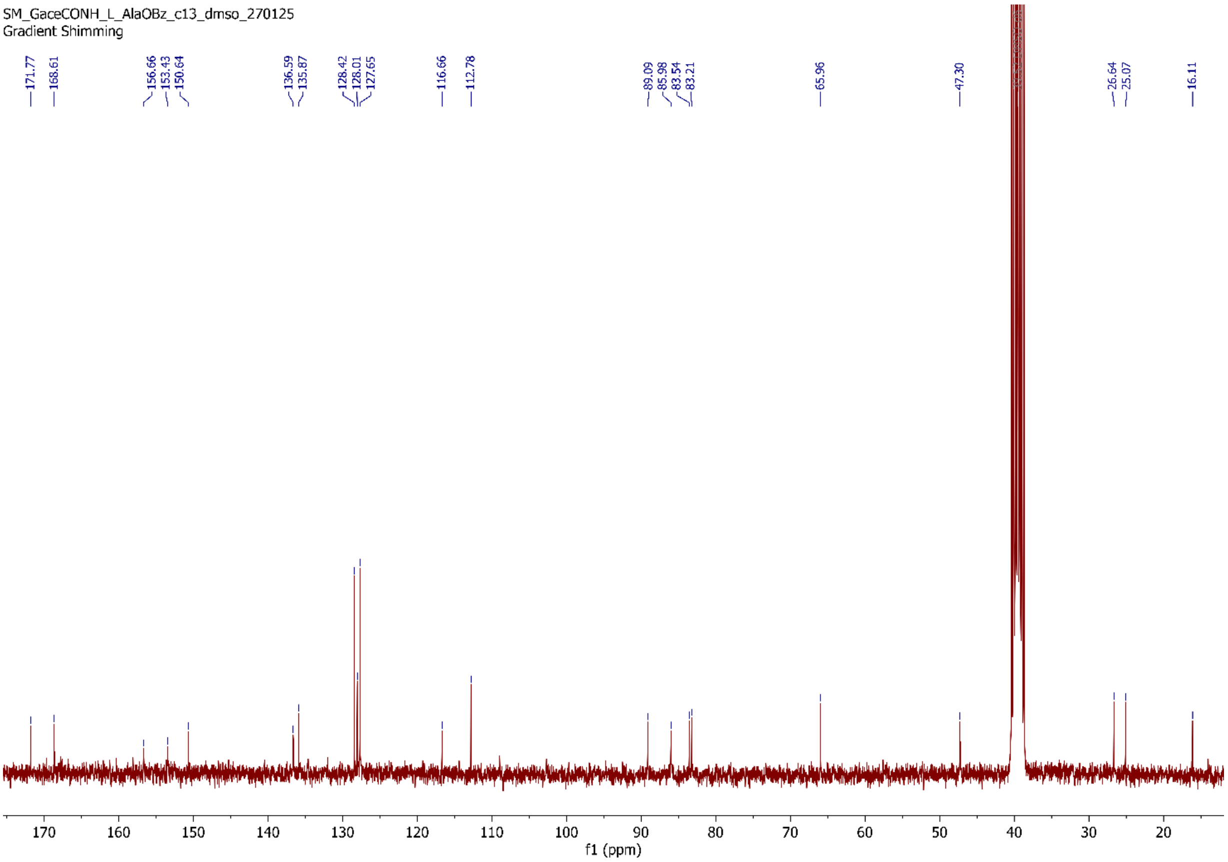

^{13}C-NMR spectrum of **2b** in DMSO-d_6

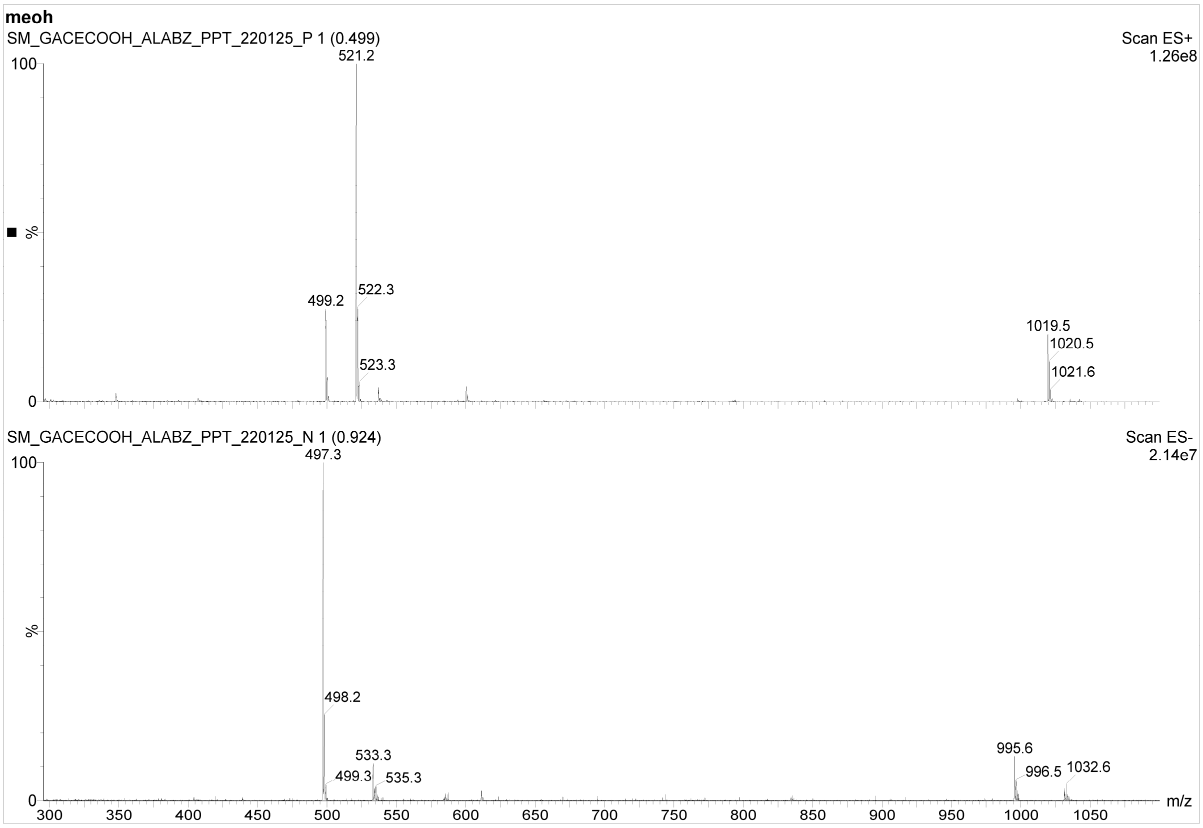

ESI-MS spectra of **2b**

2c (from D,L-alanine) (Gace_D,L_Ala_OBz)

White solid, 73% yield, CH_2Cl_2: MeOH 95:5, r.f. 0.28.

^{1}H NMR (600 MHz, DMSO-d_6) δ 10.64 (bs, 1H, guanine NH), 8.00 (D) and 7.75 (L) (d, d, J = 7.0 Hz and J = 7.3 Hz resp., 1H, -CH-NH-CO-), 7.86 (D) and 7.83 (L) (s, s, 1H, H8), 7.38 – 7.30 (m, 5H, Ph), 6.38 (bs, 2H, NH_2), 6.17 (L) and 6.12 (D) (d, d, J = 1.4 Hz and J = 1.8 Hz resp., 1H, H1'), 5.43 (L) and 5.31 (D) (dd, dd, J = 6.1, 2.4 Hz and J = 6.1, 2.6 Hz resp., 1H, H3'), 5.27 (L) and 5.21 (D) (dd, dd, J = 6.1, 1.5 Hz and J = 6.2, 1.8 Hz resp., 1H, H2'), 5.05-5.13 (m, 2H, CH_2), 4.55 (D) and 4.53 (L) (d, d, J = 2.6 Hz and J = 2.4 Hz resp., 1H, H4'), 4.20 (L) and 4.17 (D) (p, p, J = 7.2 Hz, 1H, NH-CH-CO), 1.52 and 1.32 (L) (s, s, 3H, C^{IV}-CH_3), 1.51 and 1.33 (D) (s, s, 3H, C^{IV}-CH_3), 1.22 (D) and 1.00 (L) (d, d, J = 7.2 and J = 7.3 Hz resp., 3H, CH-CH_3).

^{13}C NMR (75 MHz, DMSO-d_6) δ 171.78 (L) and 171.64 (D) (COOBz), 168.62 (L) and 168.54 (D) (5'CONH), 156.75, 156.69, 153.64, 153.50, 150.65 (C4), 136.58 (L) and 136.09 (D) (C8), 135.88 (Ph), 135.80 (Ph), 128.41 (Ph), 128.05 (Ph), 128.01 (Ph), 127.98 (Ph), 127.86 (Ph), 127.65 (Ph), 116.71 (D) and 116.67 (L) (C5), 113.08 (D) and 112.79 (L) (CMe_2), 89.10 (L) and 88.94 (D) (C1'), 86.00 (L) and 85.42 (D) (C4'), 83.71 (D) and 83.54 (L) (C2'), 83.22 (L) and 82.83 (D) (C3'), 66.12 and 65.96 (Ph-CH_2), 47.69 (D) and 47.30 (L) (CH-NH-CO), 26.74 (D) (CH_3), 26.64 (L) (CH_3), 25.09 (D+L) (CH_3), 16.87 (D) (CH-CH_3), 16.12 (L) (CH-CH_3).

HRMS (MALDI/Q-TOF) m/z: $[M + H]^+$ calcd. for $C_{23}H_{27}N_6O_7$ 499.1941, found 499.1960.

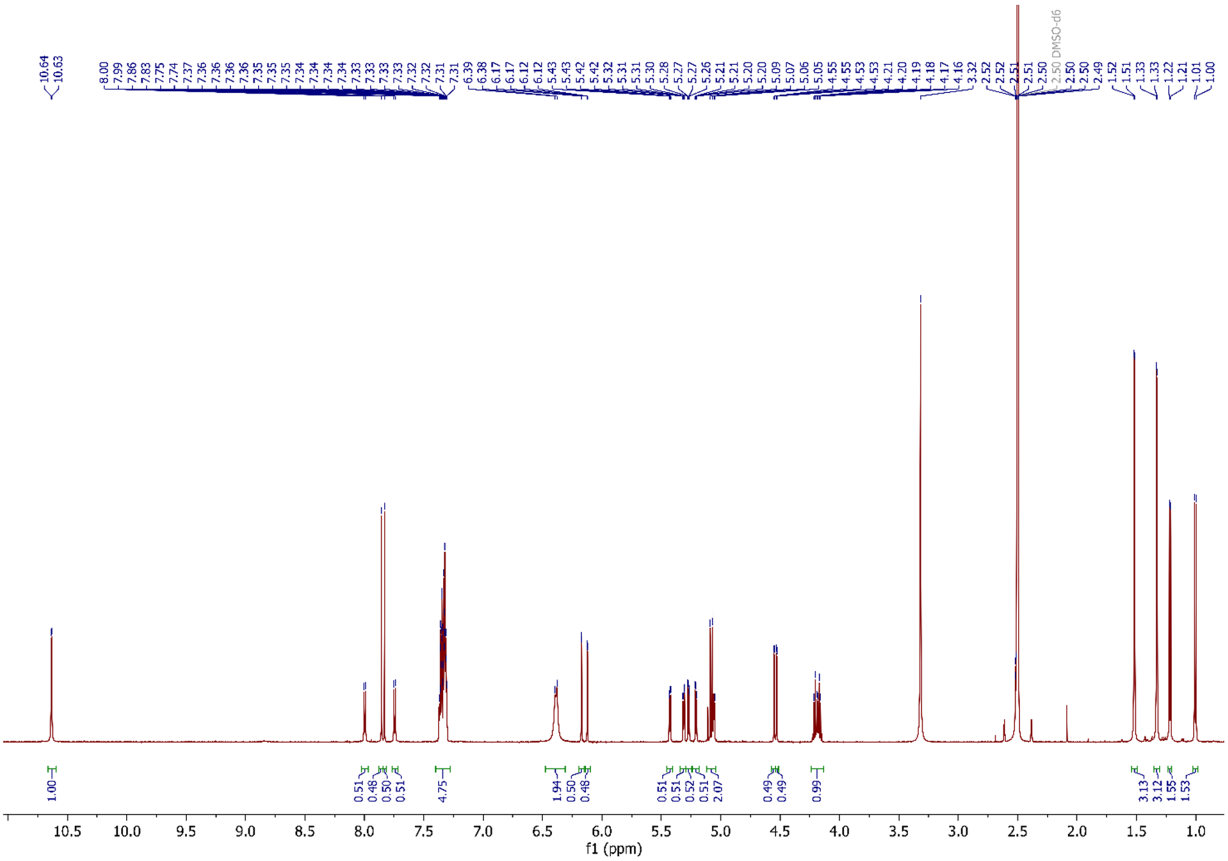

^{1}H-NMR spectrum of **2c** in DMSO-*d6*

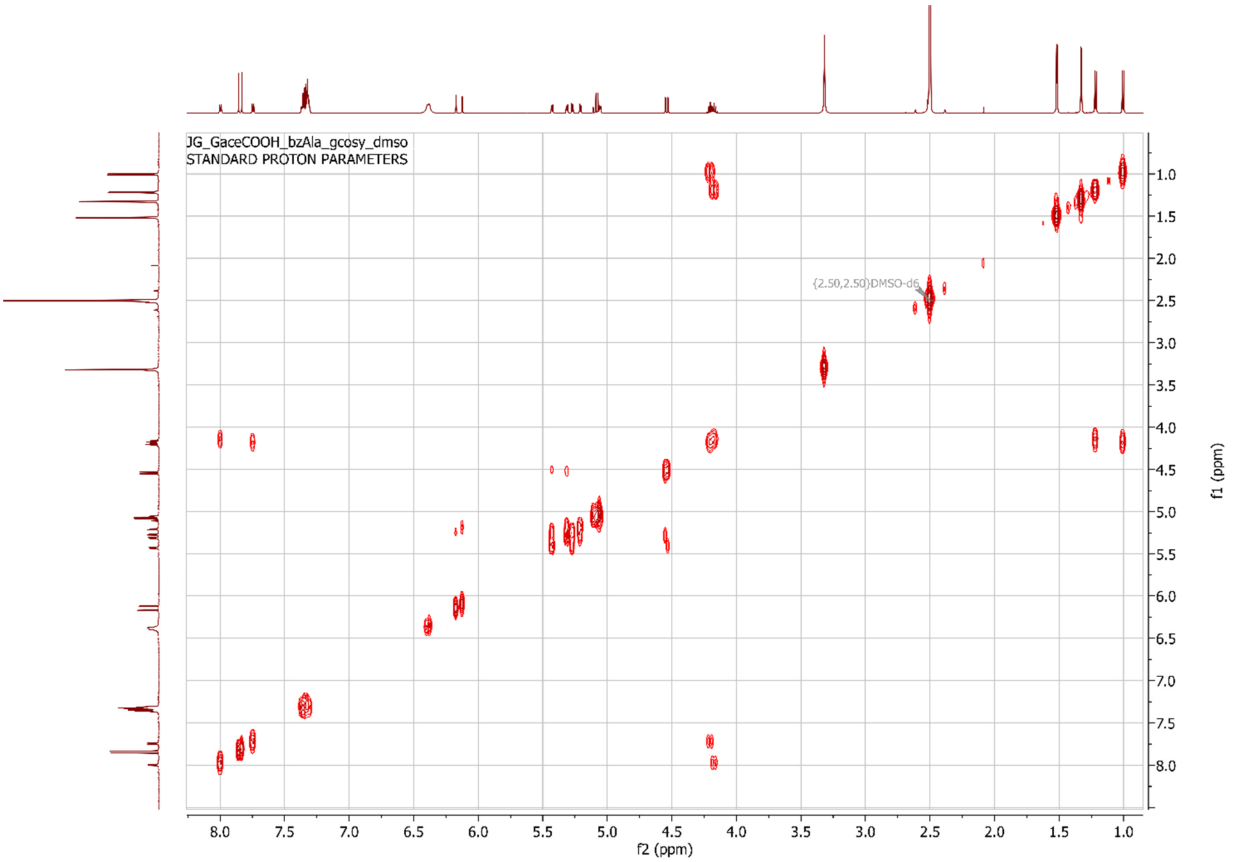

COSY spectrum of **2c** in DMSO-*d6*

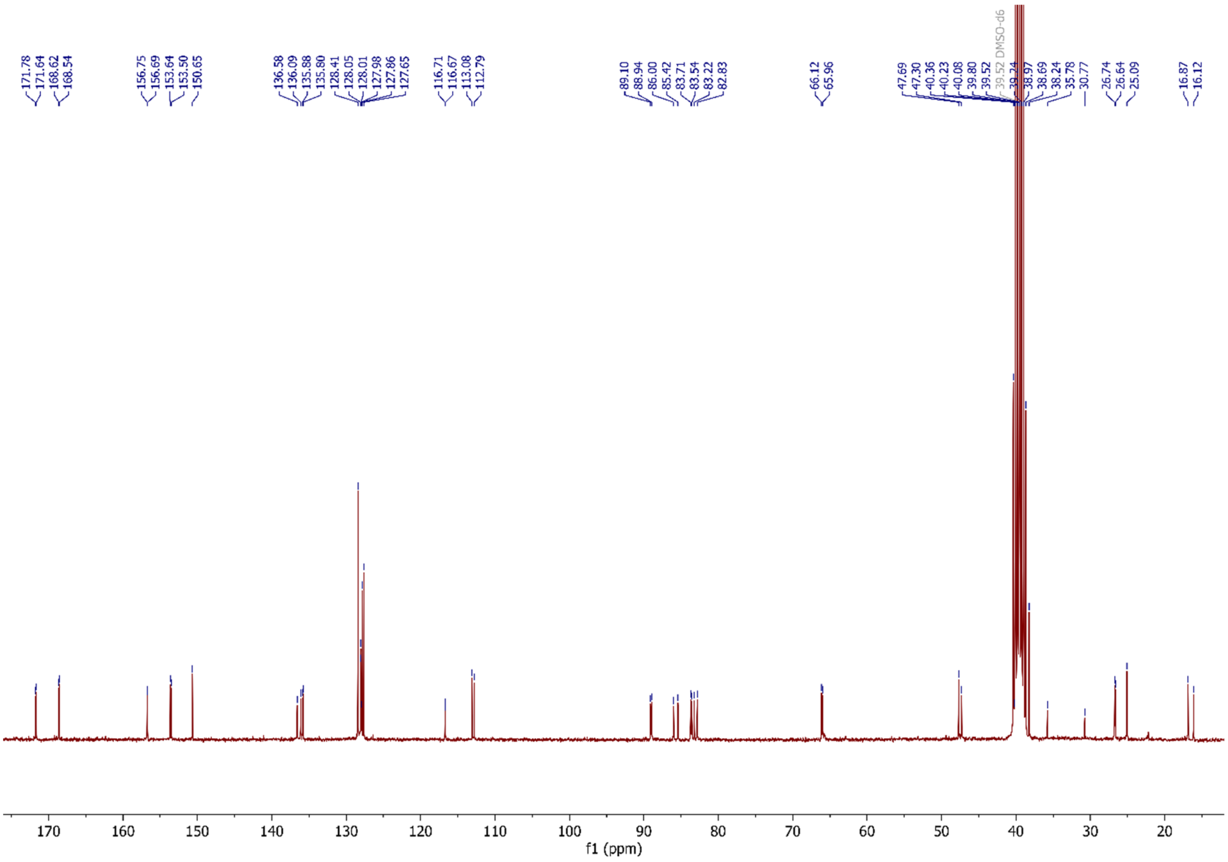

^{13}C-NMR spectrum of **2c** in DMSO-*d6*

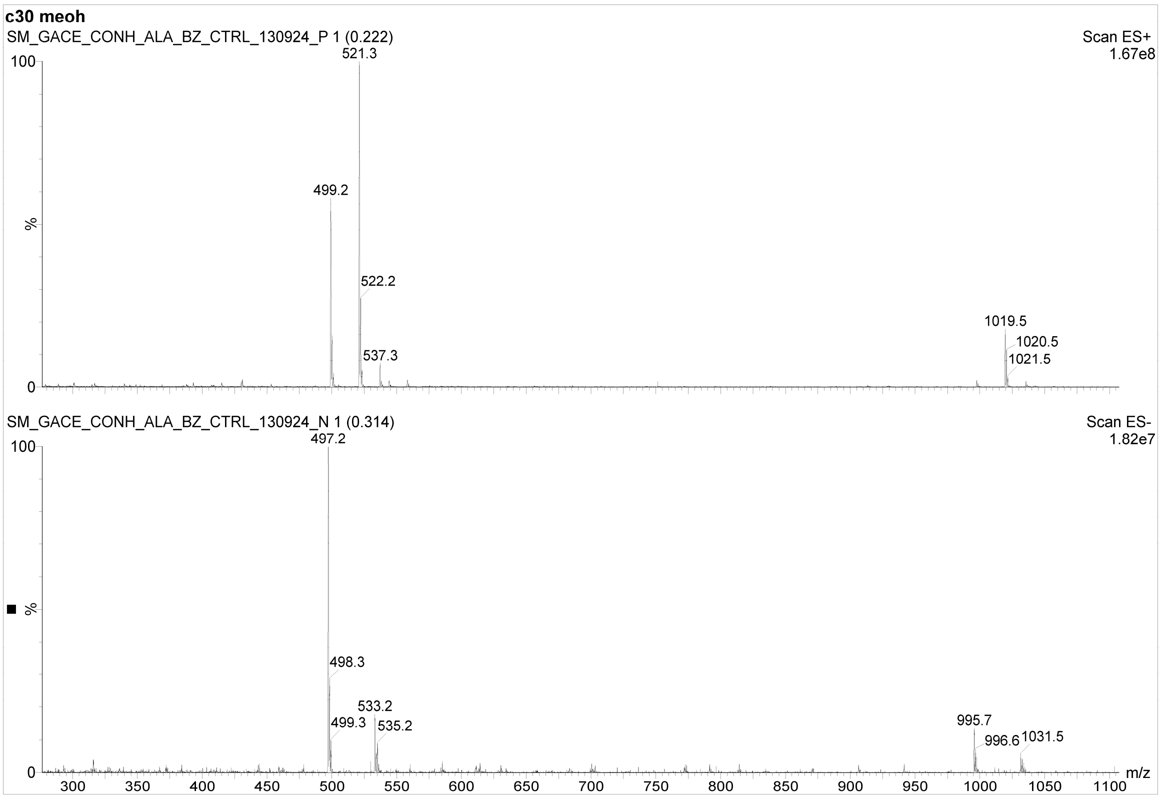

ESI-MS spectra of **2c**

1b - General procedure for the synthesis of **3** (**Gace_AA**):

A 3 necked 500 mL round bottomed flask equipped with a large magnetic stirrer was loaded with **Gace_AA_OBz** (1 mmol) MeOH (25 mL) and Pd$(OH)_2$ 20% on carbon (30 mg). The flask was purged from Oxygen by vacuum/N_2 cycles and then saturated with H_2 by vacuum/H_2 cycles. The suspension was vigorously stirred for 3-4 hrs at r.t., then fluxed with N_2 and filtered over a short celite pad. The precipitate was repeatedly washed with MeOH and hot THF. The filtrates were concentrated in vacuo to afford the product as a white solid.

3a Gace_Gly

Yield 90%

^{1}H NMR (400 MHz, DMSO-*d*$_6$) δ 10.67 (bs, 1H, guanine NH), 7.85 (s, 1H, H8), 7.77 (t, *J* = 5.7 Hz, 1H, -CH_2-NH-CO), 6.42 (bs, 2H, NH_2), 6.12 (d, *J* = 2.0 Hz, 1H, H1'), 5.30 (dd, *J* = 6.2, 2.7 Hz, 1H, H3'), 5.23 (dd, *J* = 6.3, 2.0 Hz, 1H, H2'), 4.55 (d, *J* = 2.7 Hz, 1H, H4'), 3.73 and 3.47 (dd, dd, *J* = 17.5, 6.0 Hz, 2H, CH_2), 1.52 (s, 3H, CH_3), 1.33 (s, 3H, CH_3).

^{13}C NMR (75 MHz, DMSO-*d*$_6$) δ 170.66 (COOH), 168.91 (5'CONH), 156.71, 153.68, 150.68 (C4), 136.19 (C8), 116.73 (C5), 113.21 (CMe_2), 88.87 (C1'), 85.38 (C4'), 83.40 (C2'), 82.91 (C3'), 40.35 (CH_2), 26.76 (CH_3), 25.11 (CH_3).

HRMS (MALDI/Q-TOF) m/z: $[M - H]^-$ calcd. for $C_{15}H_{17}N_6O_7$ 393.1159, found 393.1156.

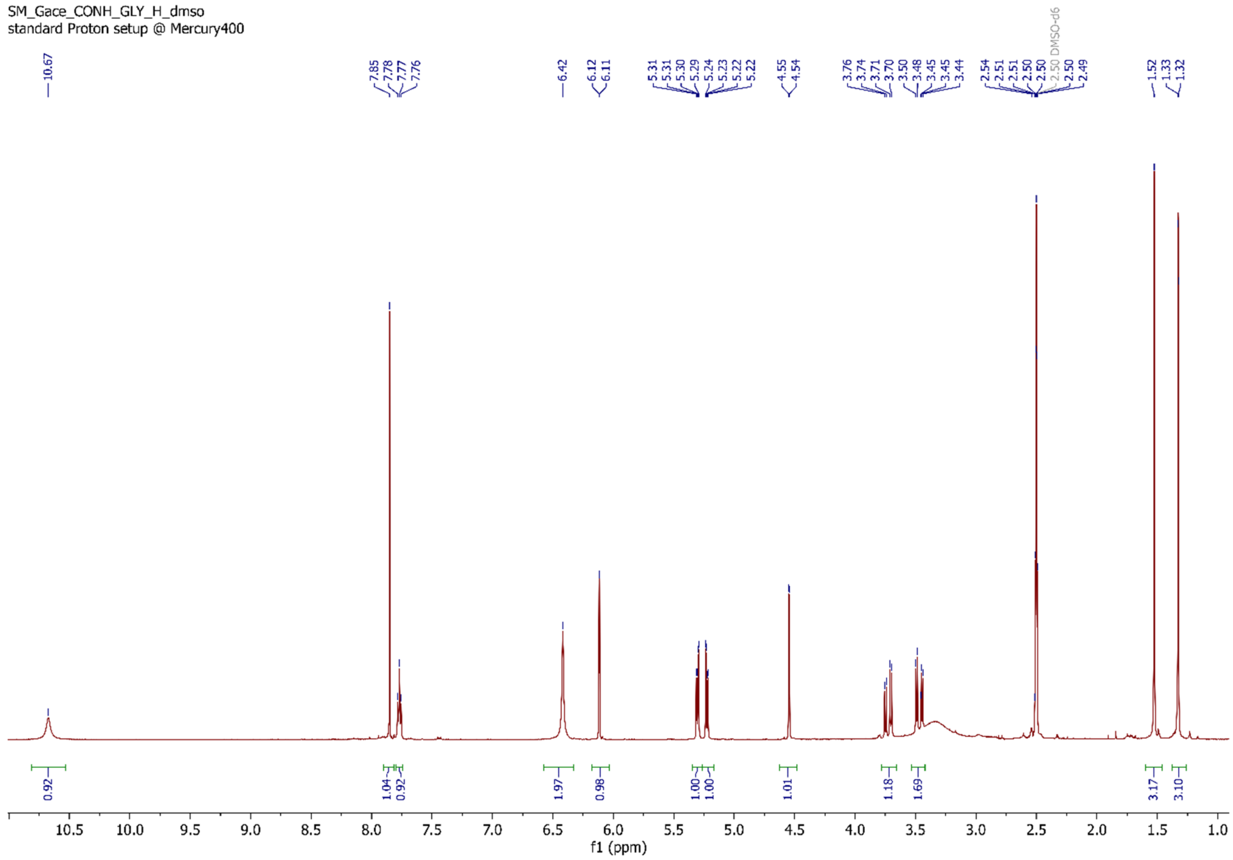

^{1}H-NMR spectrum of **3a** in DMSO-*d*$_6$

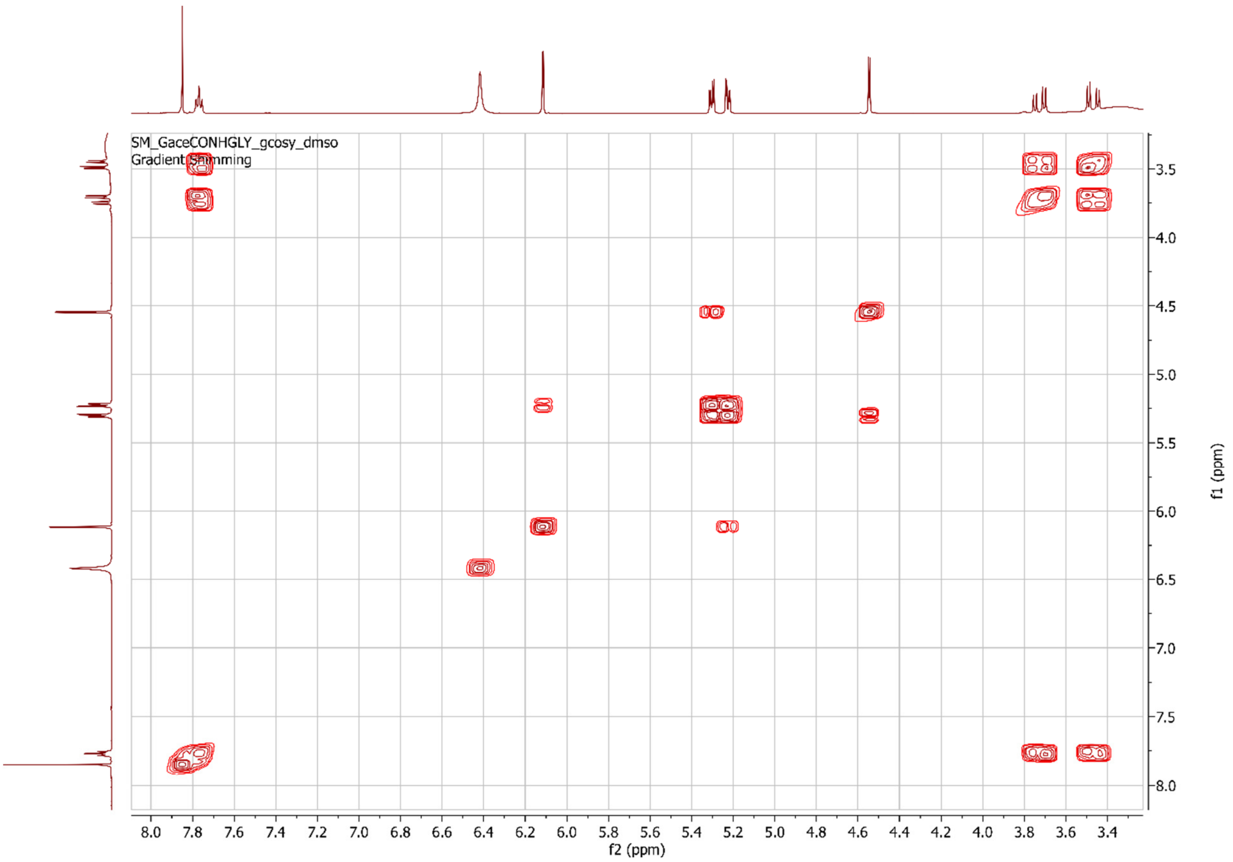

COSY spectrum of **3a** in DMSO-*d*₆

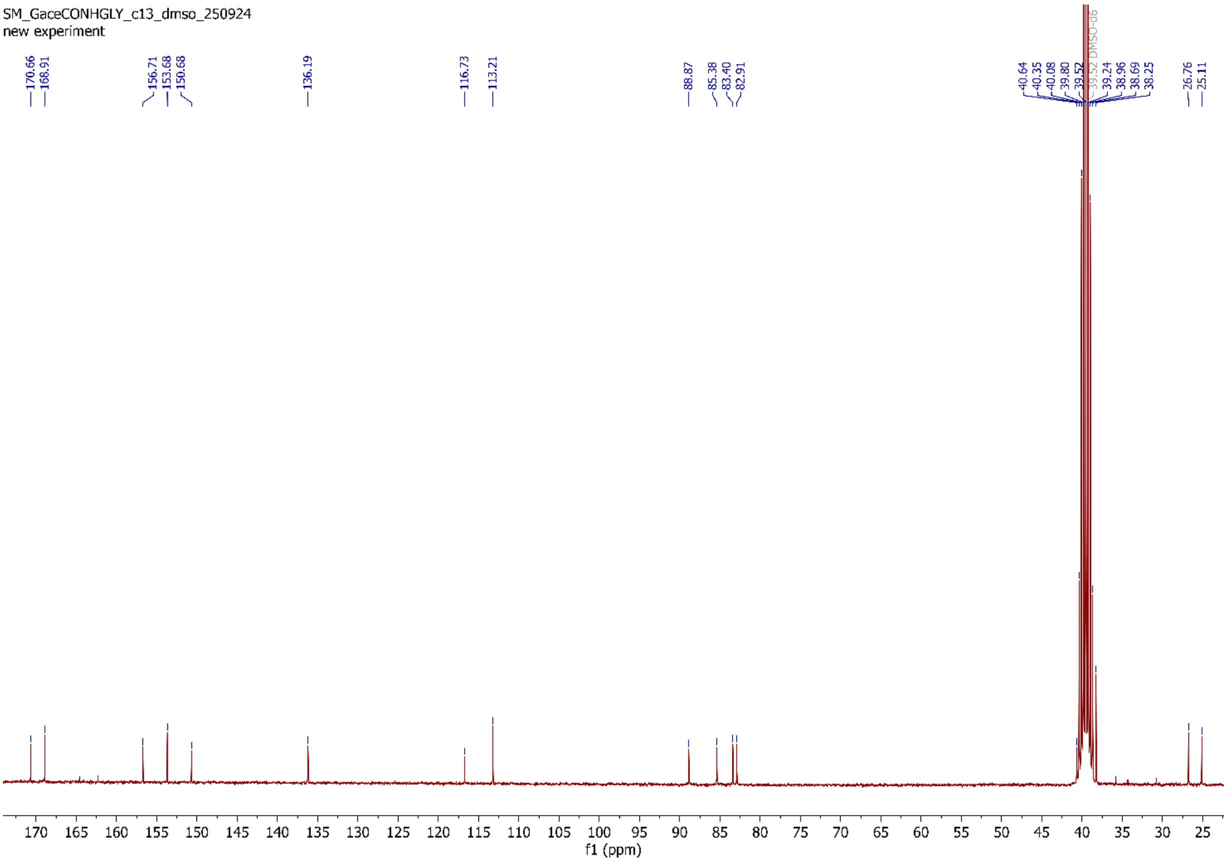

^{13}C-NMR spectrum of **3a** in DMSO-*d*₆

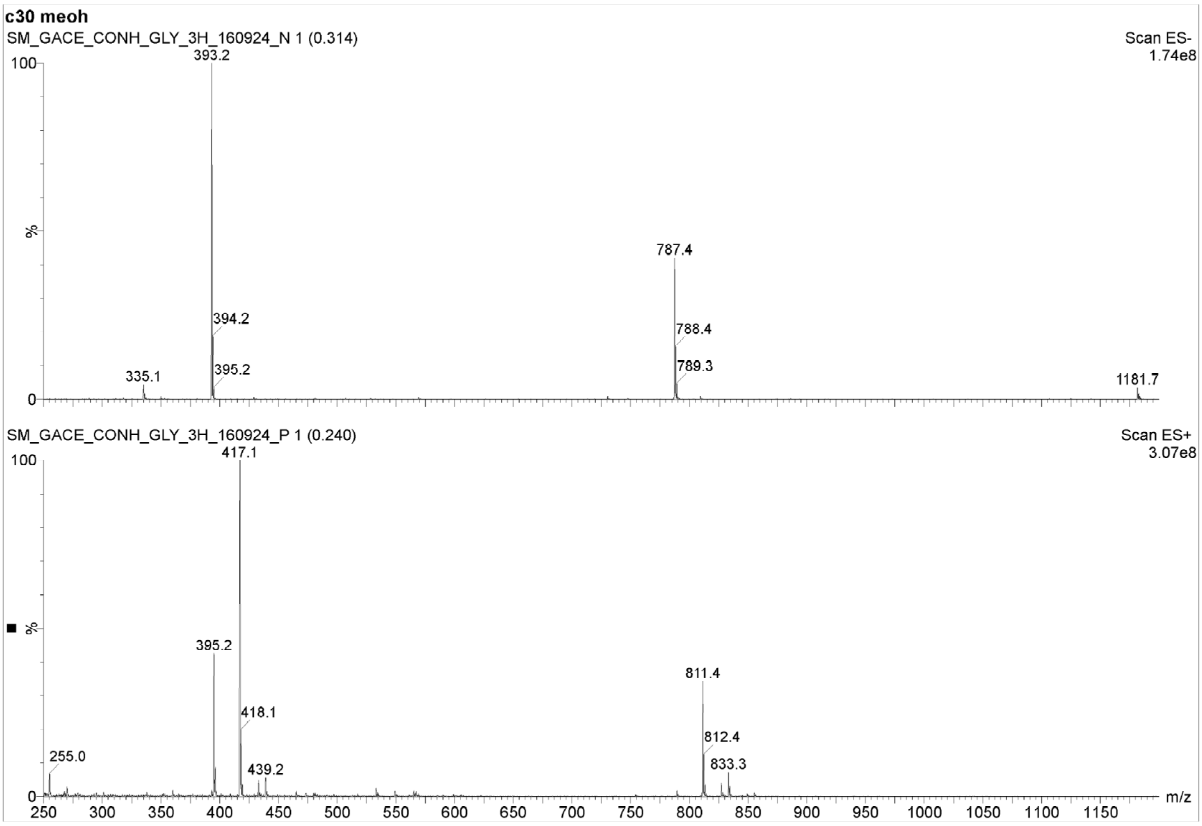

ESI-MS spectra of **3a**

3b (from L-alanine) (Gace_L_Ala)

White solid. Yield 79%

^{1}H NMR (300 MHz, DMSO-d_6) δ 12.71 (s, 1H, COOH), 10.65 (bs, 1H, guanine NH), 7.84 (s, 1H, H8), 7.36 (d, *J* = 7.2 Hz, 1H, -CH-NH-CO), 6.43 (bs, 2H, NH_2), 6.18 (d, *J* = 1.2 Hz, 1H, H1'), 5.47 (dd, *J* = 6.1, 2.3 Hz, 1H, H3'), 5.31 (dd, *J* = 6.1, 1.3 Hz, 1H, H2'), 4.53 (d, *J* = 2.3 Hz, 1H, H4'), 4.05 (p, *J* = 7.2 Hz, 1H, NH-CH-CO), 1.51 (s, 3H, C^{IV}-CH_3), 1.34 (s, 3H, C^{IV}-CH_3), 0.94 (d, *J* = 7.2 Hz, 3H, CH-CH_3).

^{13}C NMR (75 MHz, DMSO-d_6) δ 173.41 (COOH), 168.24 (5'CONH), 156.69, 153.50, 150.64 (C4), 136.56 (C8), 116.70 (C5), 112.70 (CMe_2), 89.06 (C1'), 86.20 (C4'), 83.48 (C2'), 83.22 (C3'), 47.08 (NH-CH-CO), 26.62 (CH_3), 25.08 (CH_3), 16.52 (CH-CH_3).

HRMS (MALDI/Q-TOF) m/z: $[M - H]^-$ calcd. for $C_{16}H_{19}N_6O_7$ 407.1315, found 407.1311.

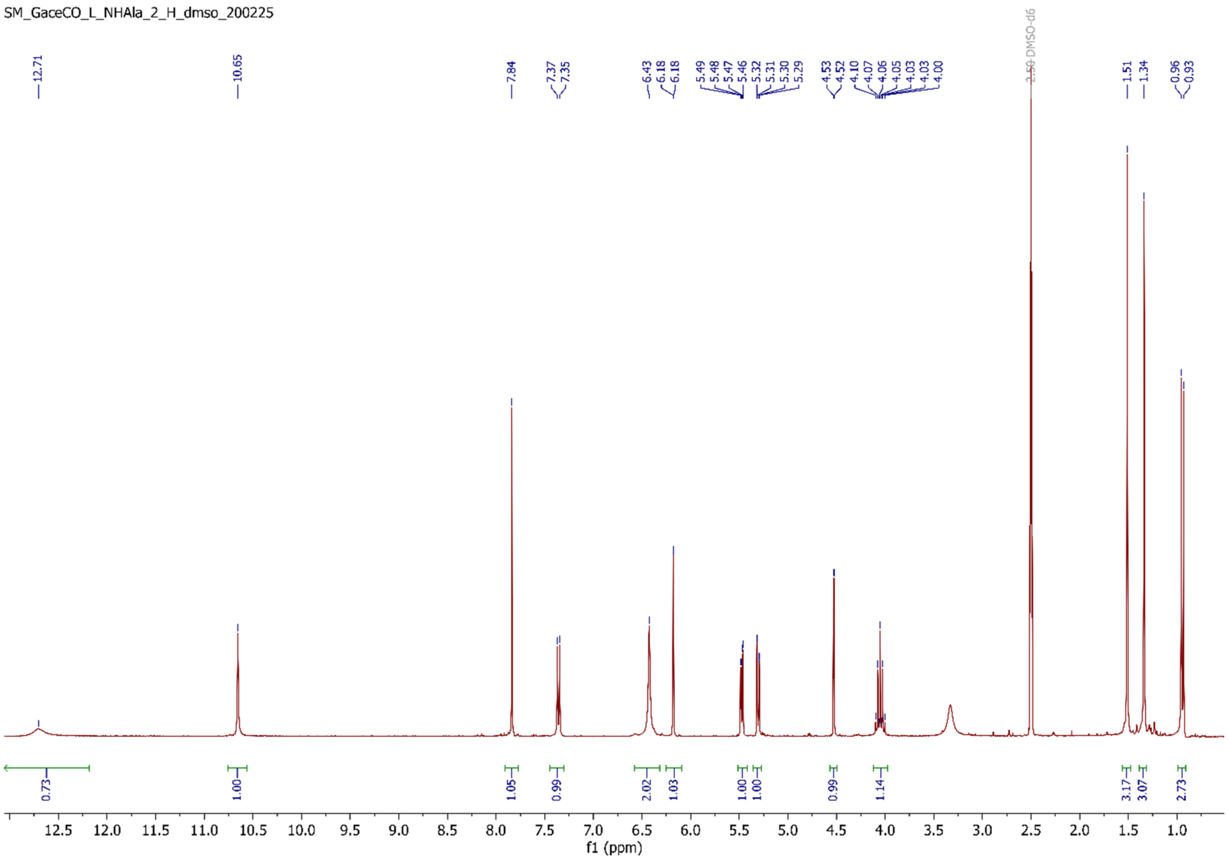

^{1}H-NMR spectrum of **3b** in DMSO-d_6

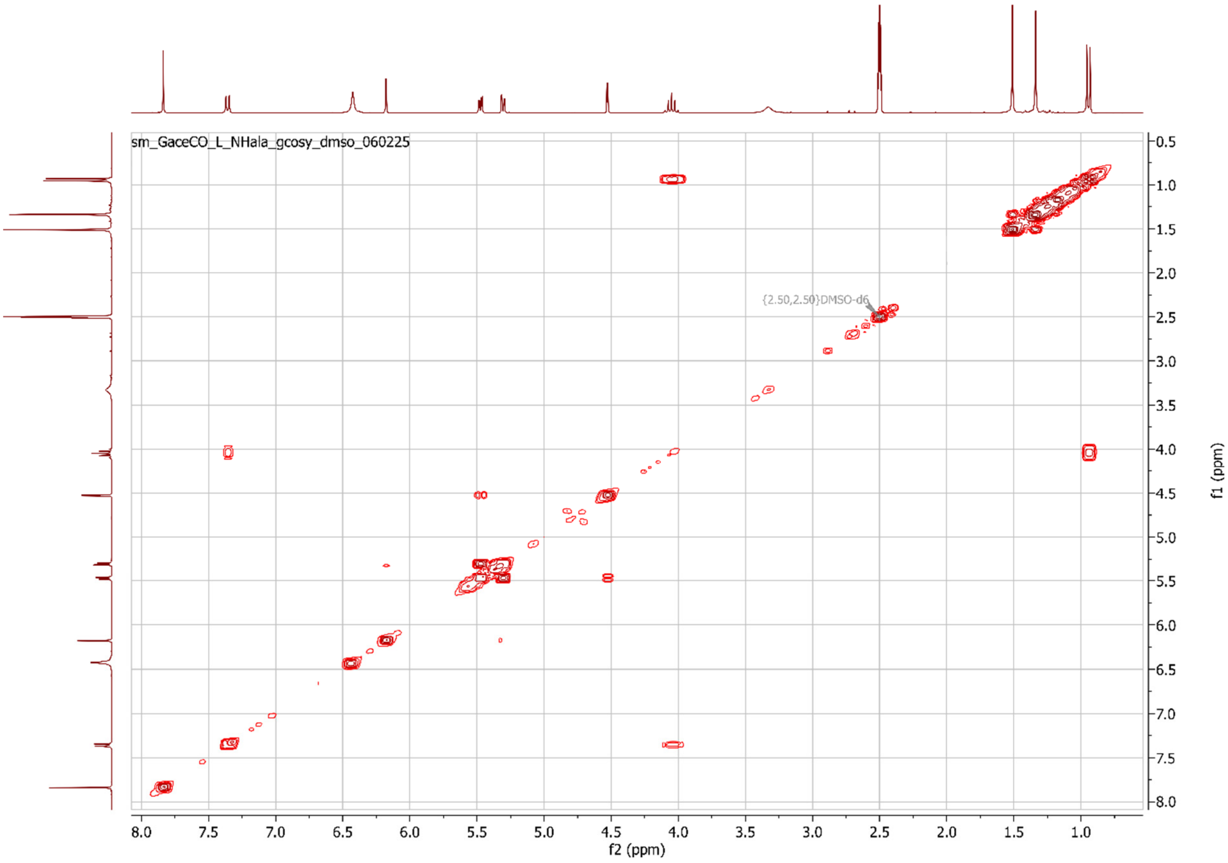

COSY spectrum of **3b** in DMSO-d_6

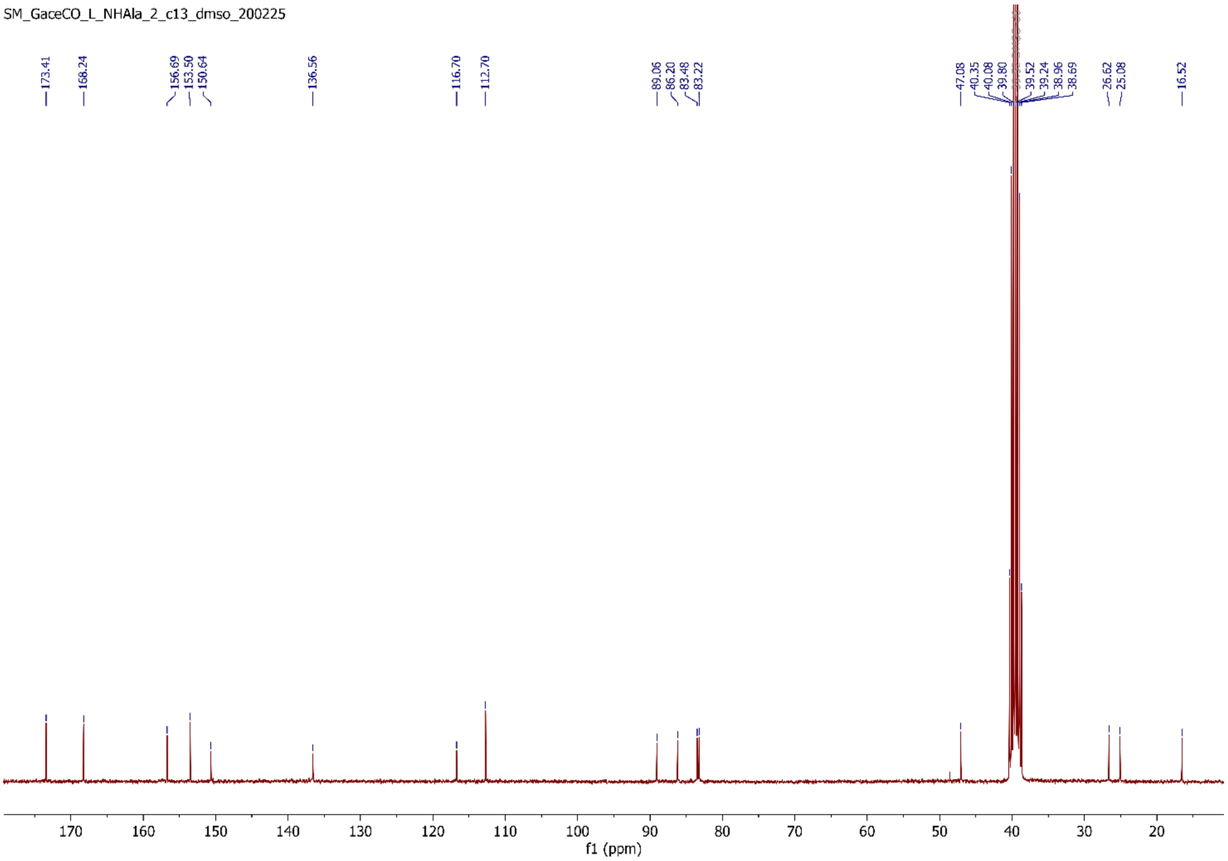

^{13}C-NMR spectrum of **3b** in DMSO-d_6

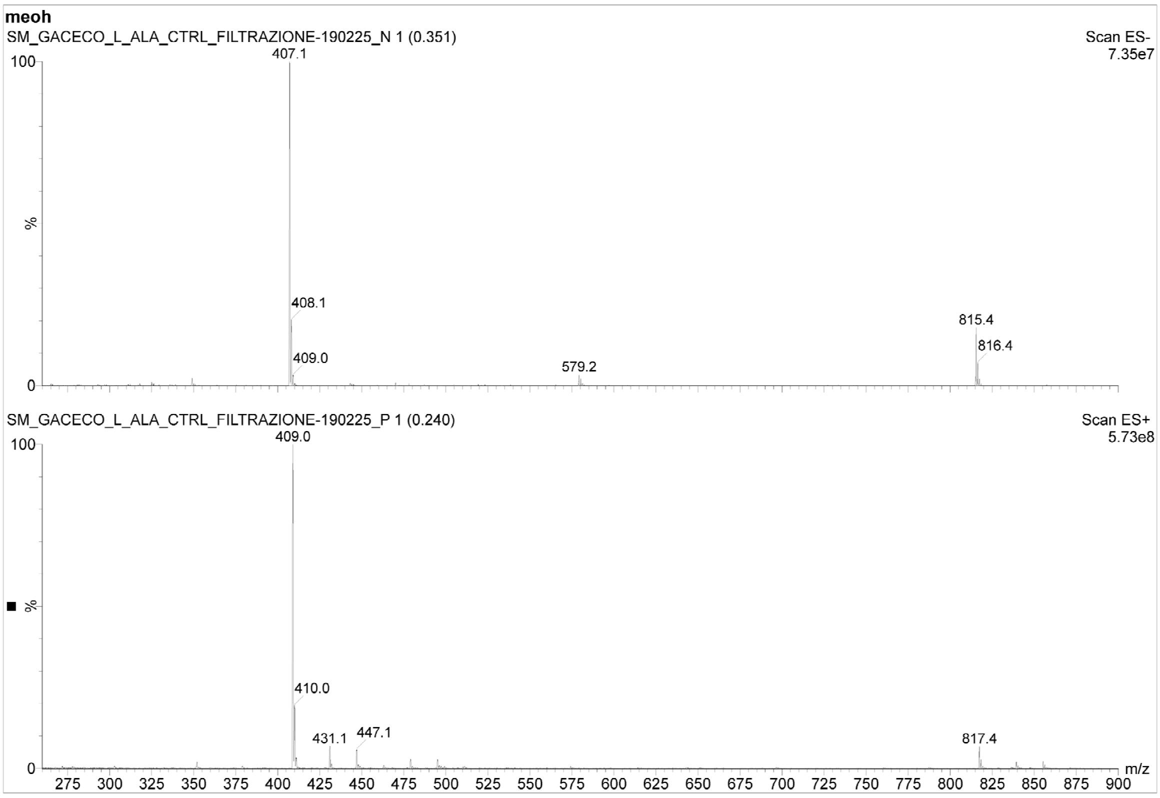

ESI-MS spectra of **3b**

3c (from D,L-alanine) (**Gace_D,L_Ala**)

White solid. Yield 87%

^{1}H NMR (300 MHz, DMSO-d_6) δ 10.81 (bs, 1H, guanine NH), 7.87 (D) and 7.83 (L) (s, s, 1H, H8), 7.81 (D) and 7.33 (d, d, *J* = 6.9 Hz and *J* = 7.1 Hz resp., 1H, -CH-N<u>H</u>-CO), 6.54 and 6.51 (bs, bs, 2H, NH_2), 6.17 (L) and 6.10 (D) (d, d, *J* = 1.2 Hz, *J* = 2.1 Hz resp., 1H, H1'), 5.47 (L) and 5.27 (D) (dd, dd, *J* = 6.1, 2.3 Hz and *J* = 6.2, 2.7 Hz resp., 1H, H3'), 5.31 (L) and 5.19 (D) (dd, dd, *J* = 5.9, 1.3 Hz and *J* = 6.2, 2.1 Hz resp., 1H, H2'), 4.54 (D) and 4.52 (L) (d, d, *J* = 2.7 Hz and *J* = 2.3 Hz resp., 1H, H4'), 4.10-3.96 (m, *J* = 7.3 Hz, 1H, NH-C<u>H</u>-CO), 1.52 (D) (s, 3H, C^{IV}-C<u>H</u>3), 1.51 (L) (s, 3H, C^{IV}-C<u>H</u>3), 1.34 (L) (s, 3H, C^{IV}-C<u>H</u>3), 1.32 (D) (s, 3H, C^{IV}-C<u>H</u>3), 1.21 (D) and 0.93 (L) (d, d, *J* = 7.2 Hz, 3H, CH-C<u>H</u>3).

^{13}C NMR (75 MHz, DMSO-d_6) δ 173.67 (D) and 173.52 (L) (COOH), 168.28 (D) and 168.13 (L) (5'<u>C</u>ONH), 156.74 (D+L), 153.78 (D) and 153.60 (L), 150.65 (L) and 150.56 (D) (C4), 136.48 (L) and 136.05 (D) (C8), 116.82 (D) and 116.70 (L) (C5), 113.24 (D) and 112.69 (L) (<u>C</u>Me_2), 89.05 (D+L) (C1'), 86.20 (L) and 85.34 (D) (C4'), 83.59 (D) and 83.45 (L) (C2'), 83.22 (L) and 82.80 (D) (C3'), 47.73 (D) and 47.26 (L) (NH-<u>C</u>H-CO), 26.82 (D) and 26.62 (L) (CH_3), 25.14 (D) and 25.08 (L) (CH_3), 17.48 (D) and 16.68 (L) (CH-<u>C</u>H_3).

HRMS (MALDI/Q-TOF) m/z: $[M - H]^-$ calcd. for $C_{16}H_{19}N_6O_7$ 407.1315, found 407.1307.

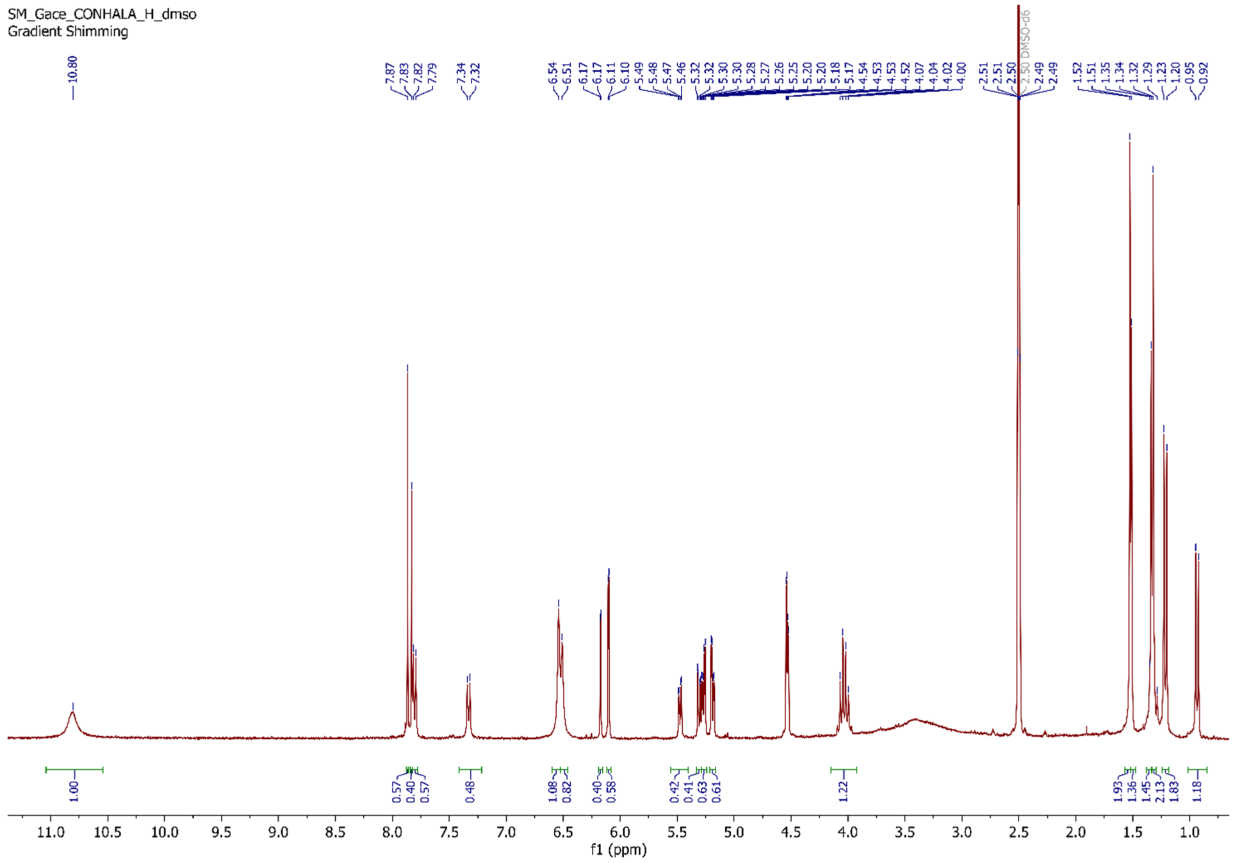

^{1}H-NMR spectrum of **3c** in DMSO-d_6

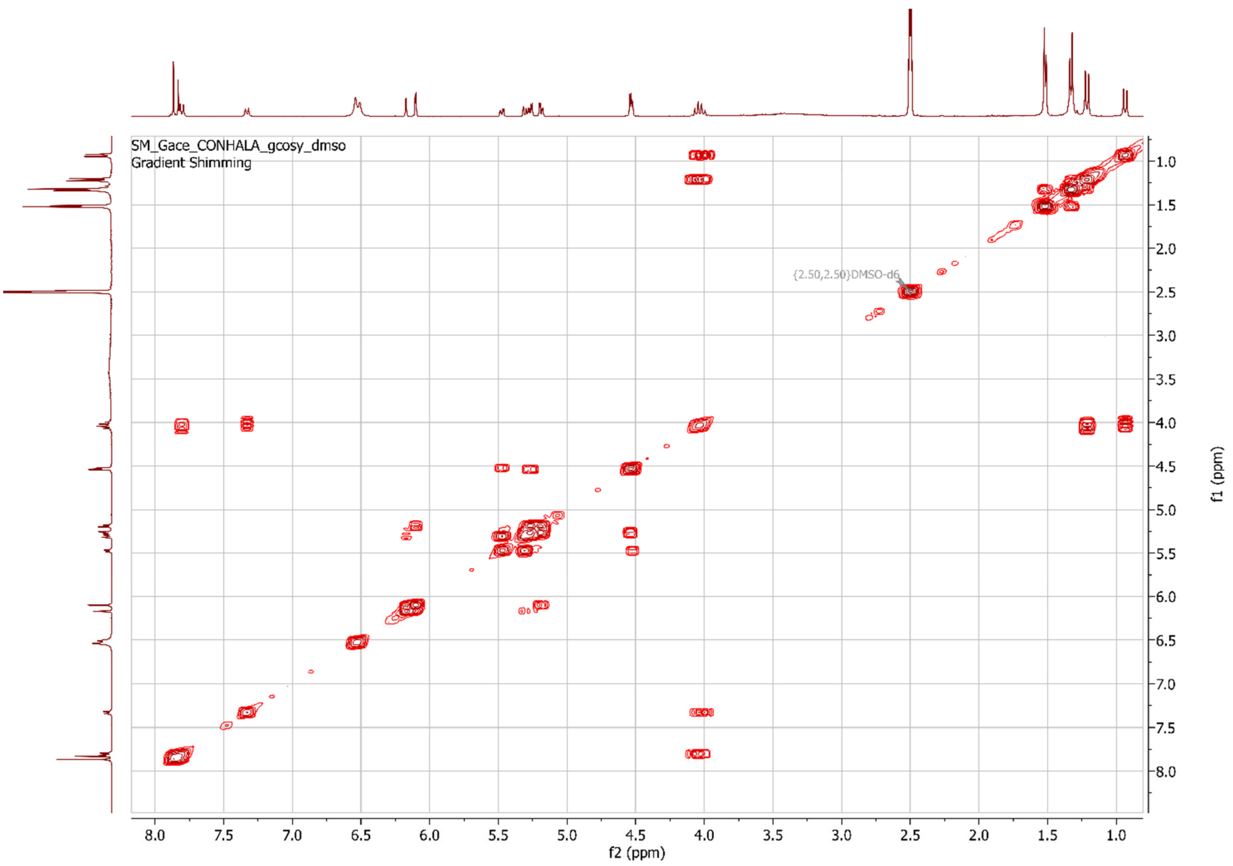

COSY spectrum of **3c** in DMSO-d_6

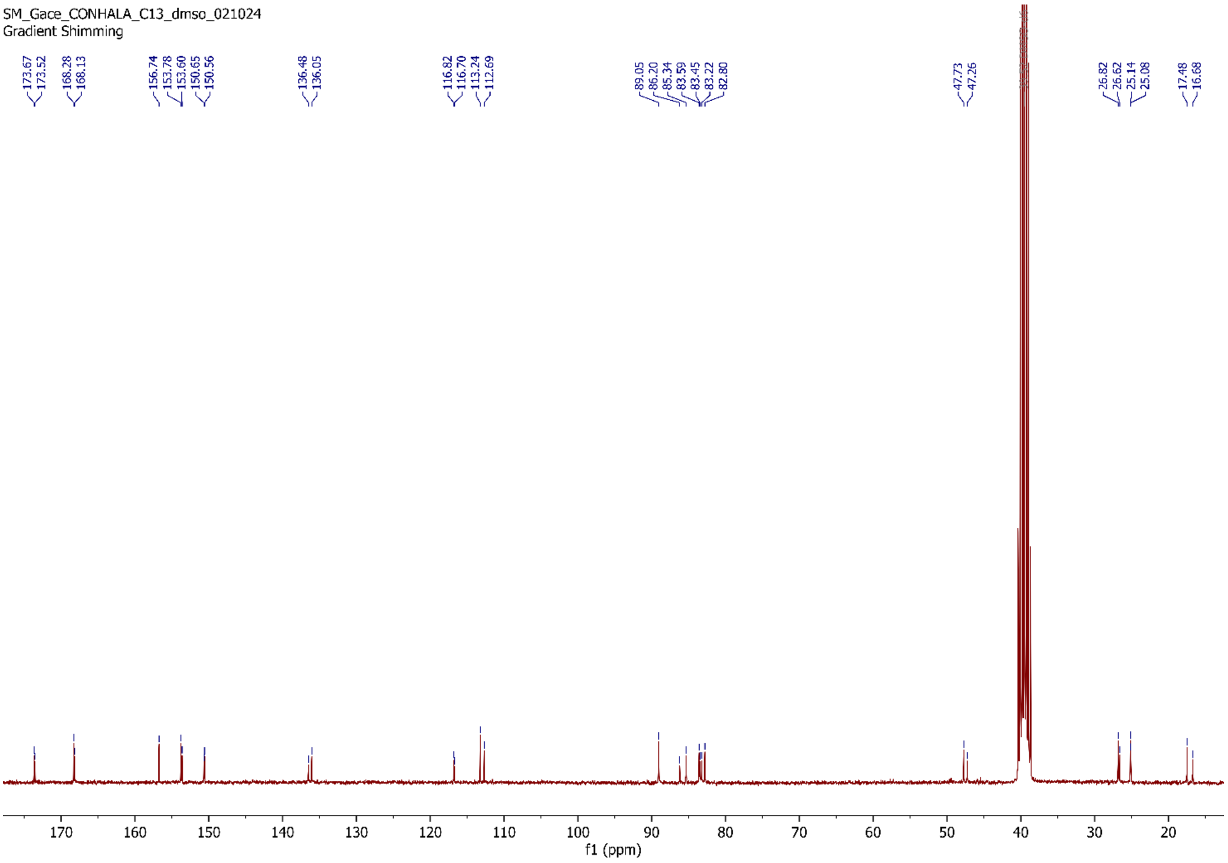

^{13}C-NMR spectrum of **3c** in DMSO-d_6

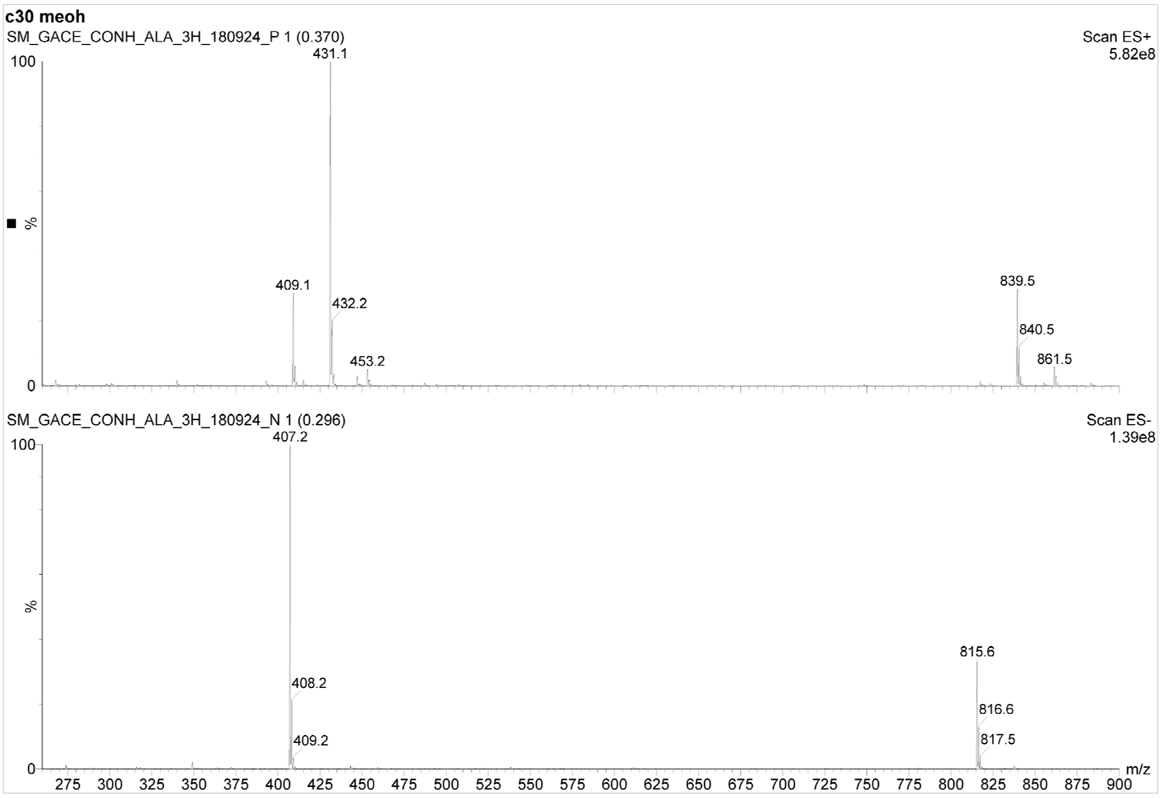

ESI-MS spectra of **3c**

1c - General procedure for the synthesis of **G_AA**):

Gace_AA (0.60 mmol) was dissolved in a 1:1 mixture of H_2O and formic acid (20 mL) and heated at 70°C with stirring. The reaction was monitored by ESI-MS and stopped as soon as all of the starting material had disappeared (2.5-3.5 hrs). The mixture was then cooled to r.t. and concentrated in vacuo. Chloroform was added to the residue and the mixture was concentrated in vacuo for 3 times. The product was crystallized from water.

G-Gly

White solid, 92% yield.

^{1}H NMR (300 MHz, DMSO-*d6*): δ 10.70 (bs, 1H, guanine NH), 8.32 (t, *J* = 6.0 Hz, 1H, -CH2-NH-CO-), 8.02 (s, 1H, H8), 6.48 (bs, 2H, NH_2), 5.84 (d, *J* = 7.7 Hz, 1H, H1'), 4.51 (dd, *J* = 7.6, 4.5 Hz, 1H, H2'), 4.34 (d, *J* = 1.6 Hz, 1H, H4'), 4.18 (dd, *J* = 4.5, 1.6 Hz, 1H, H3'), 3.94 – 3.72 (m, 2H, CH_2).

^{13}C NMR (75 MHz, DMSO-*d6*) δ 171.21 (COOH), 169.82 (5'CONH), 156.71, 153.83, 151.59 (C4), 135.97 (C8), 116.80 (C5), 85.85 (C1'), 83.70 (C4'), 73.11 (C3'), 72.63 (C2'), 40.67 (CH_2).

HRMS (MALDI/Q-TOF) m/z: $[M - H]^-$ calcd. for $C_{12}H_{13}N_6O_7$ 353.0846, found 353.0851.

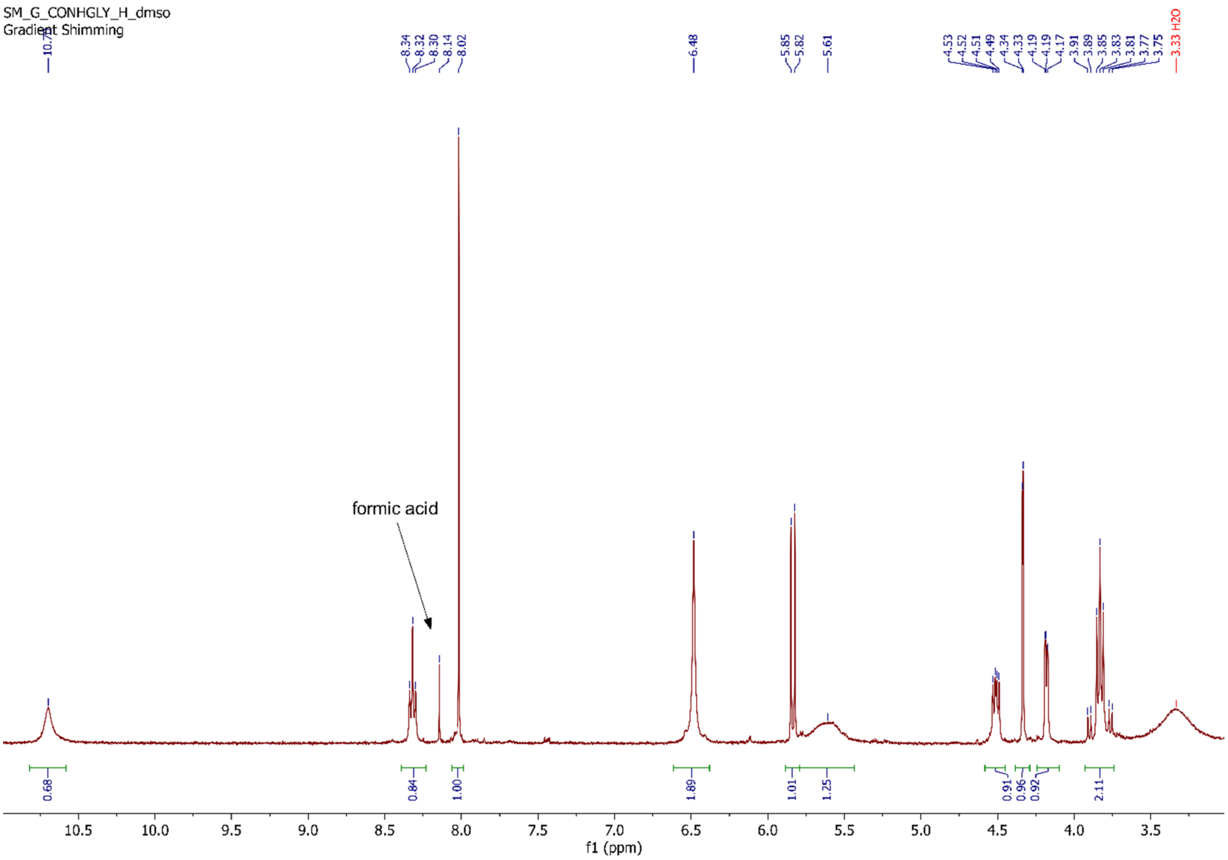

^{1}H-NMR spectrum of **G-Gly** in DMSO-d_6

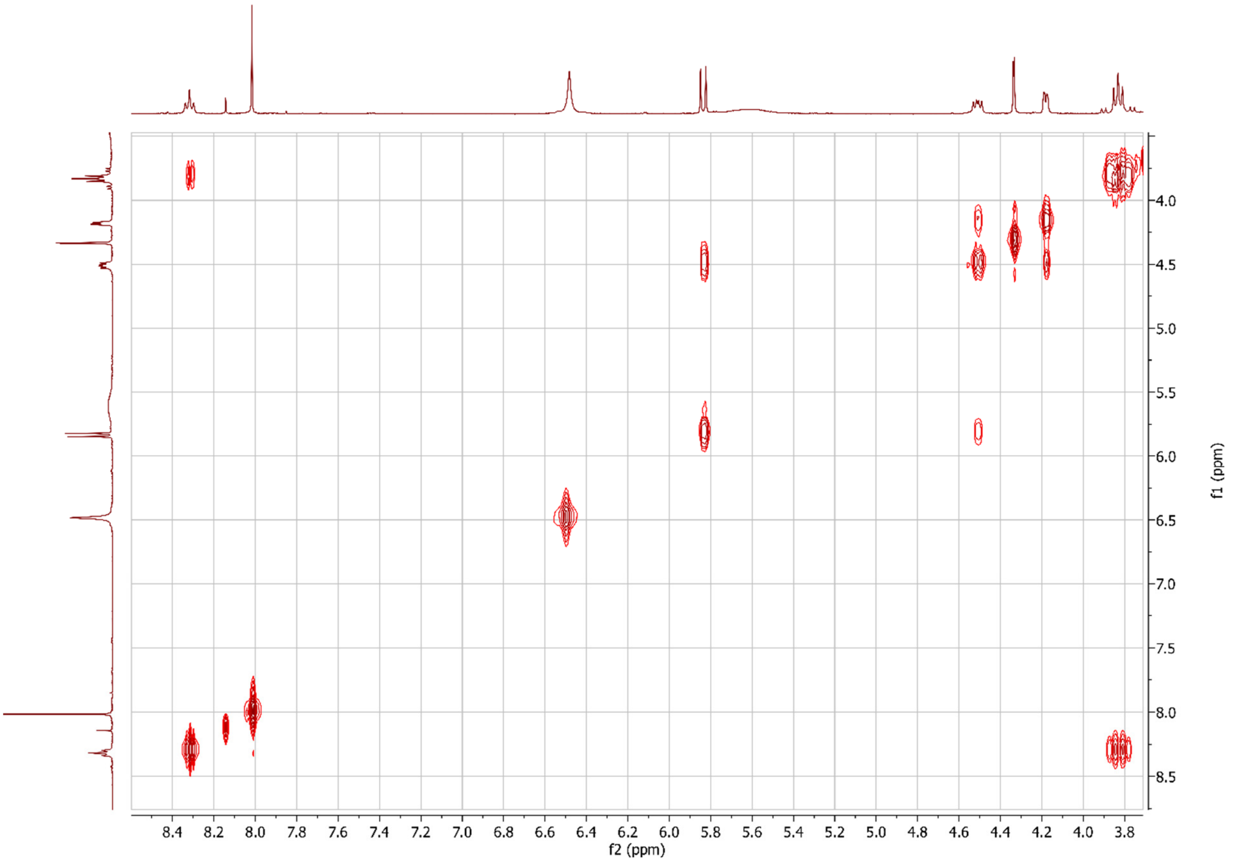

COSY spectrum of **G-Gly** in DMSO-d_6

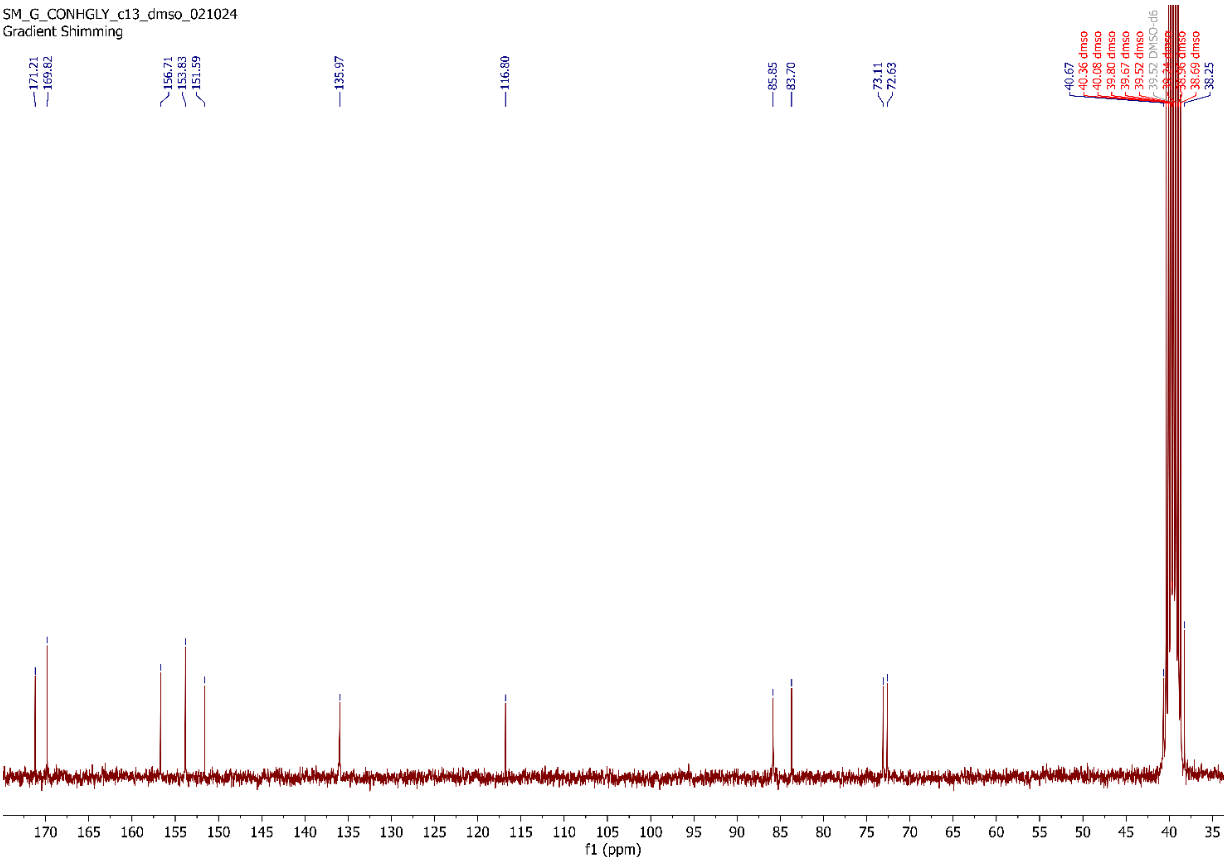

^{13}C-NMR spectrum of **G-Gly** in DMSO-*d₆*

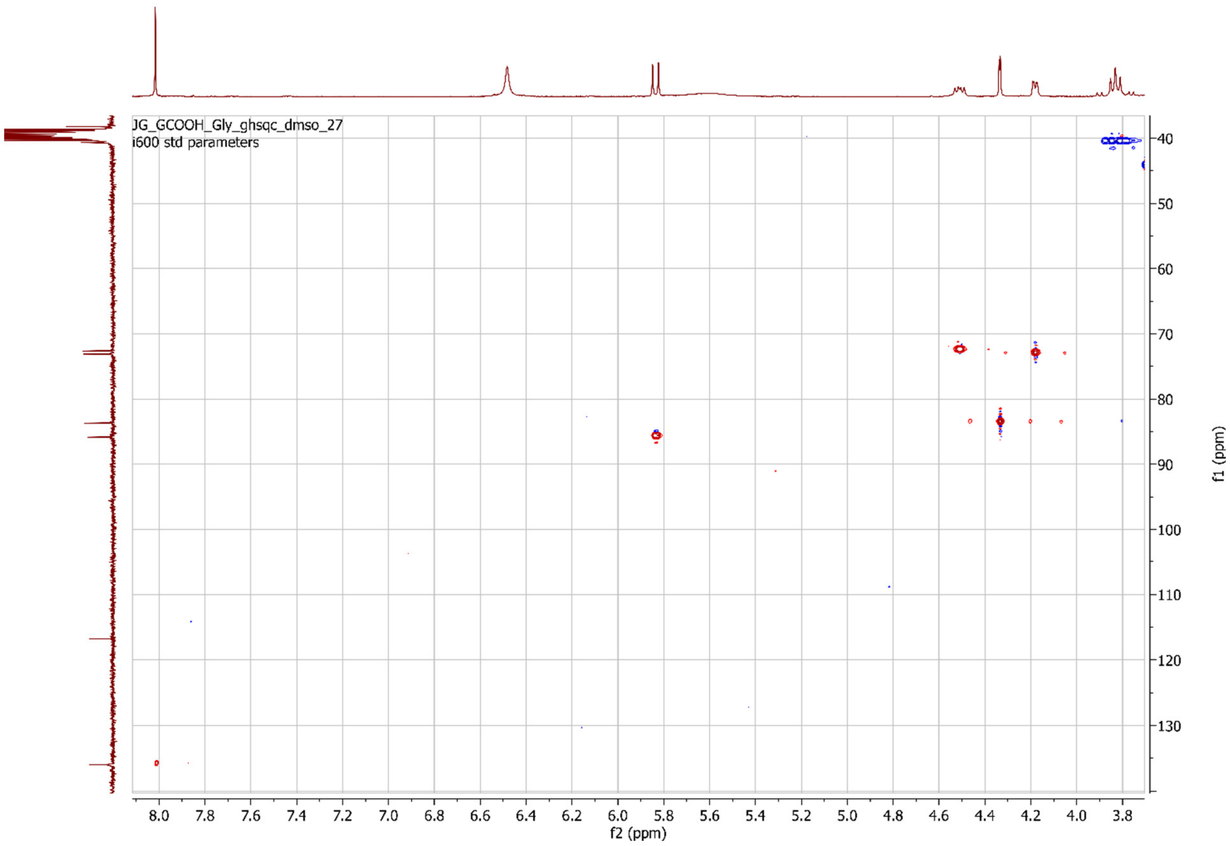

HSQC spectrum of **G-Gly** in DMSO-d_6

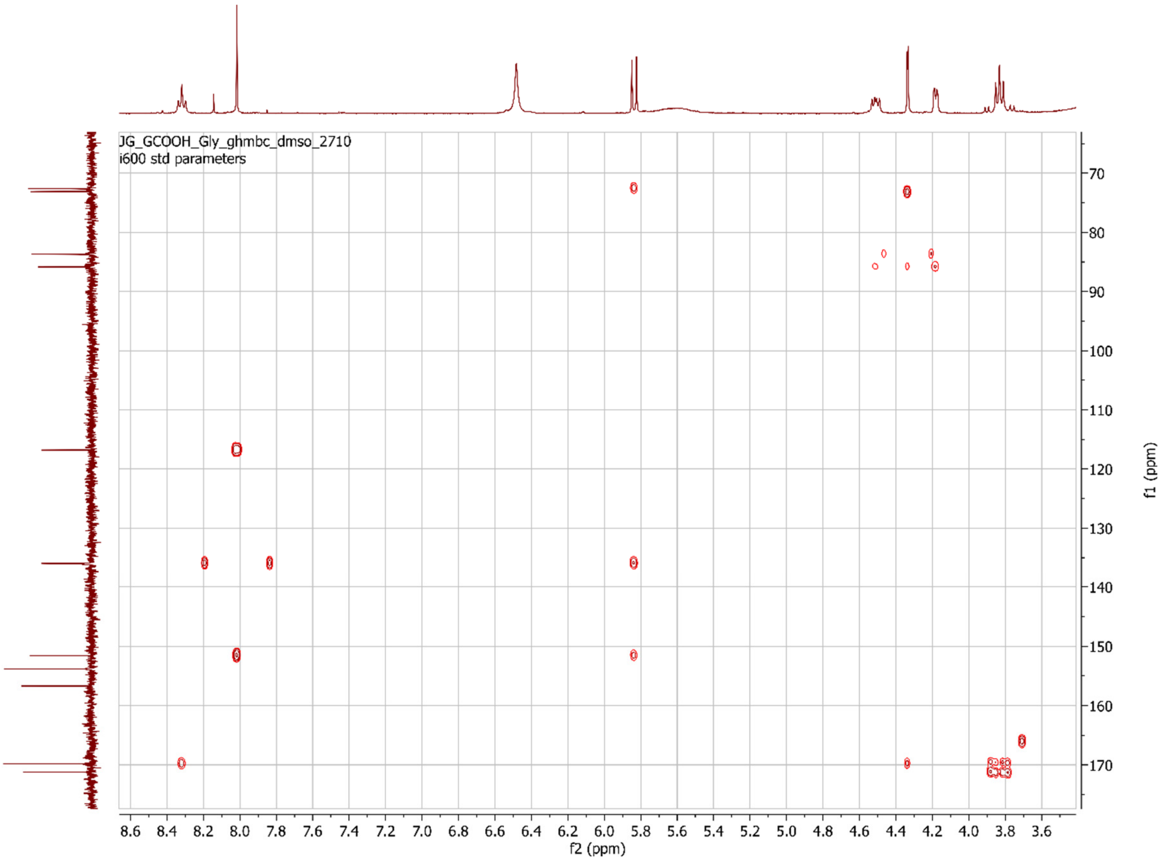

HMBC spectrum of **G-Gly** in DMSO-d_6

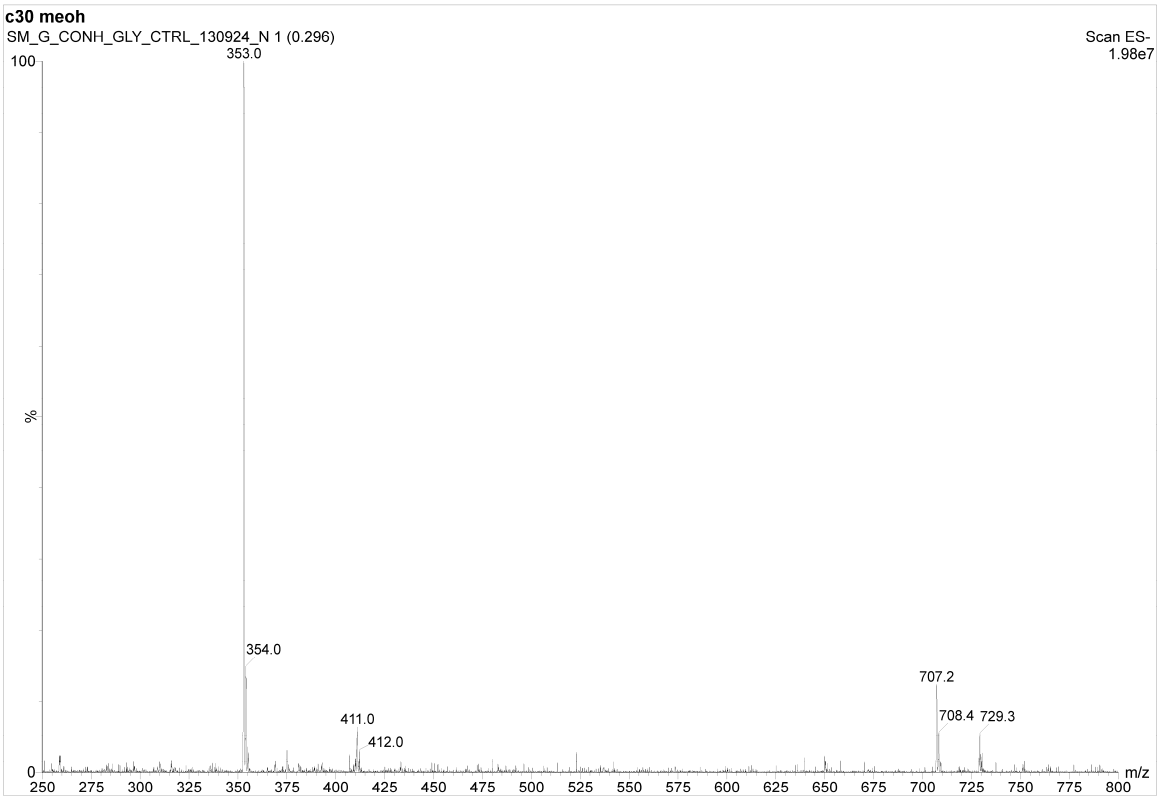

ESI-MS spectrum of **G-Gly**

G-L-Ala

White solid, 87% yield.

^{1}H NMR (300 MHz, DMSO-d_6) δ 12.66 (bs, 1H, COOH), 10.65 (bs, 1H, guanine NH), 8.31 (d, J = 7.2 Hz, 1H, -CH-NH-CO-), 8.06 (s, 1H, H8), 6.48 (bs, 2H, NH_2), 5.82 (d, J = 7.3 Hz, 1H, H1'), 5.57 (bs, 2H, OH), 4.46-4.58 (m, 1H, H2'), 4.36 (d, J = 1.7 Hz, 1H, H4'), 4.25 (p, J = 7.3 Hz, 1H, NH-CH-CO), 4.18 – 4.11 (m, 1H, H3'), 1.30 (d, J = 7.3 Hz, 3H, CH_3).

^{13}C NMR (75 MHz, DMSO-d_6) δ 173.69 (COOH), 169.46 (5'CONH), 156.73, 153.78, 151.76 (C4), 135.60 (C8), 116.56 (C5), 85.80 (C1'), 83.45 (C4'), 73.55 (C2'), 73.06 (C3'), 47.53 (NH-CH-CO), 16.93 (CH_3).

HRMS (MALDI/Q-TOF) m/z: $[M - H]^-$ calcd. for $C_{13}H_{15}N_6O_7$ 367.1002, found 367.0996.

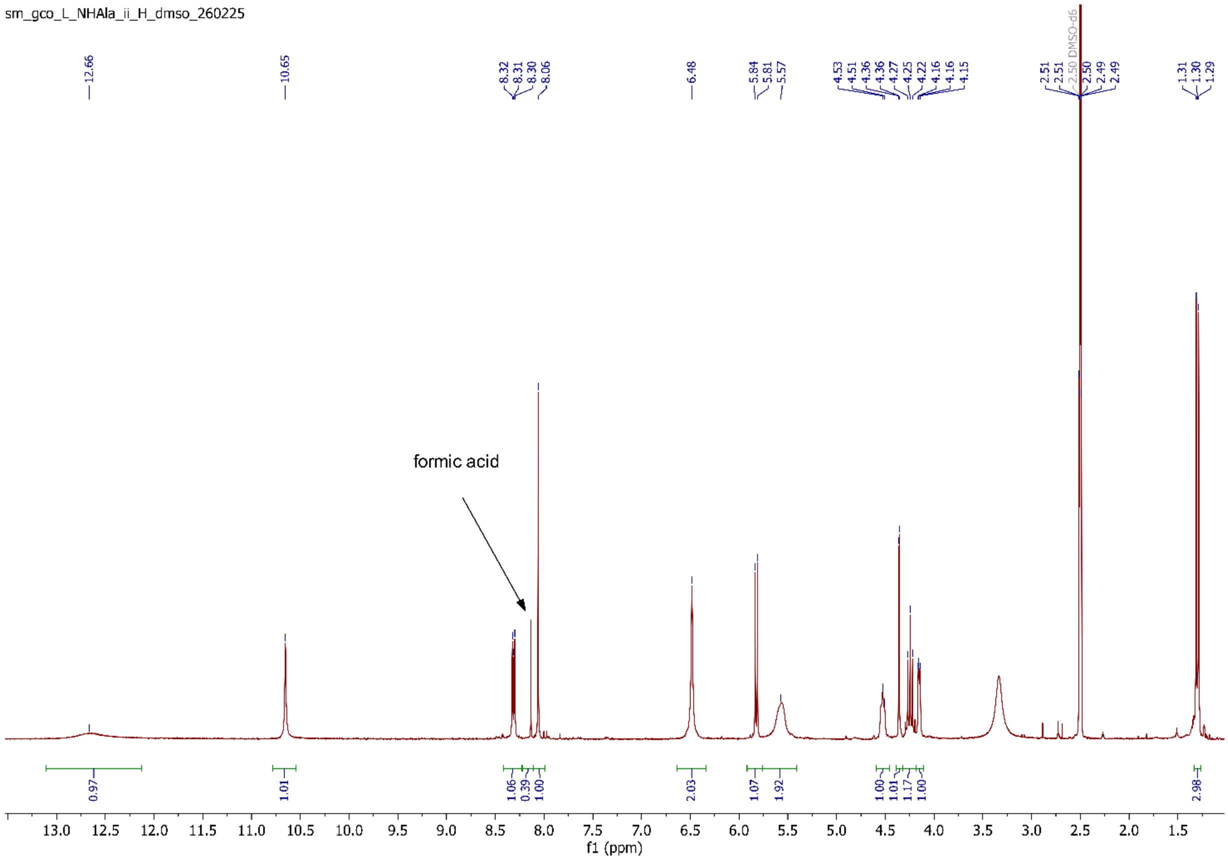

^{1}H-NMR spectrum of **G-L-Ala** in DMSO-d_6

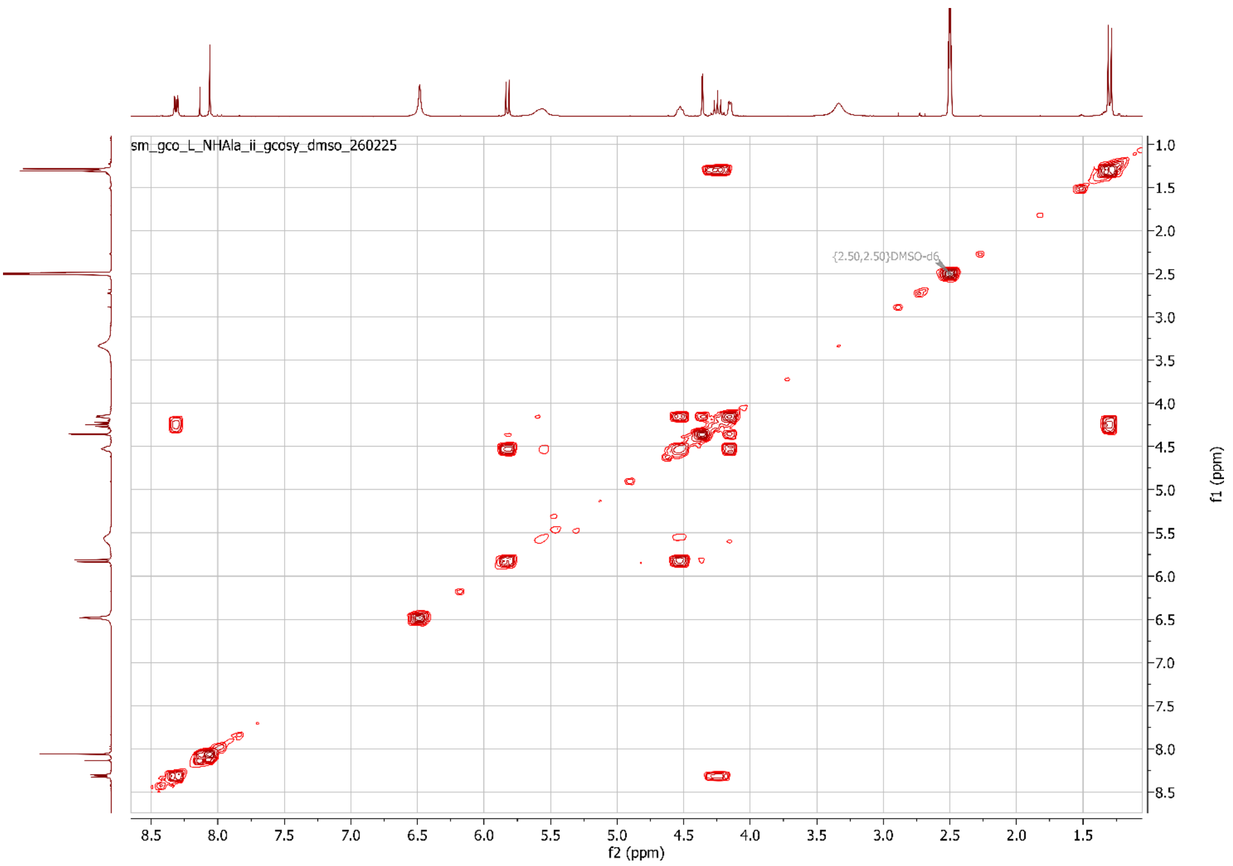

COSY spectrum of **G-L-Ala** in DMSO-d_6

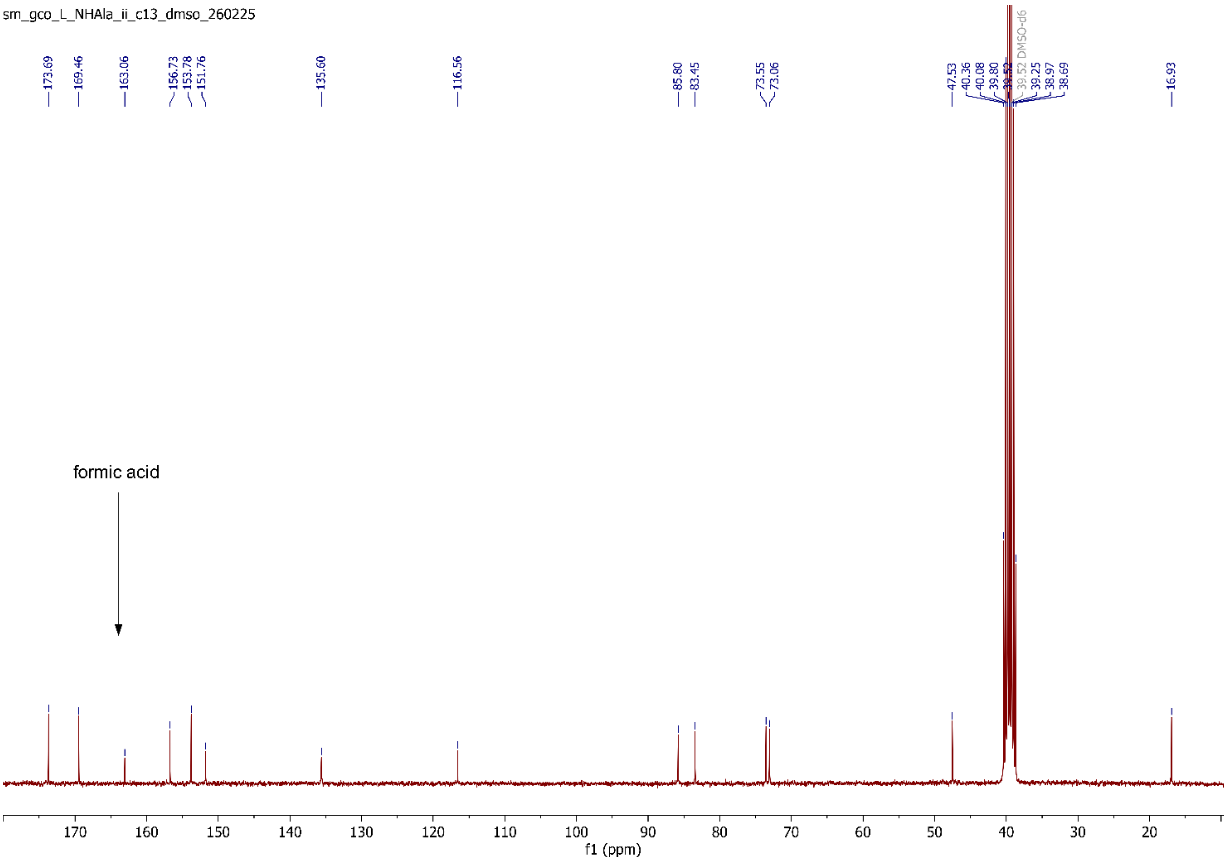

^{13}C-NMR spectrum of **G-L-Ala** in DMSO-d_6

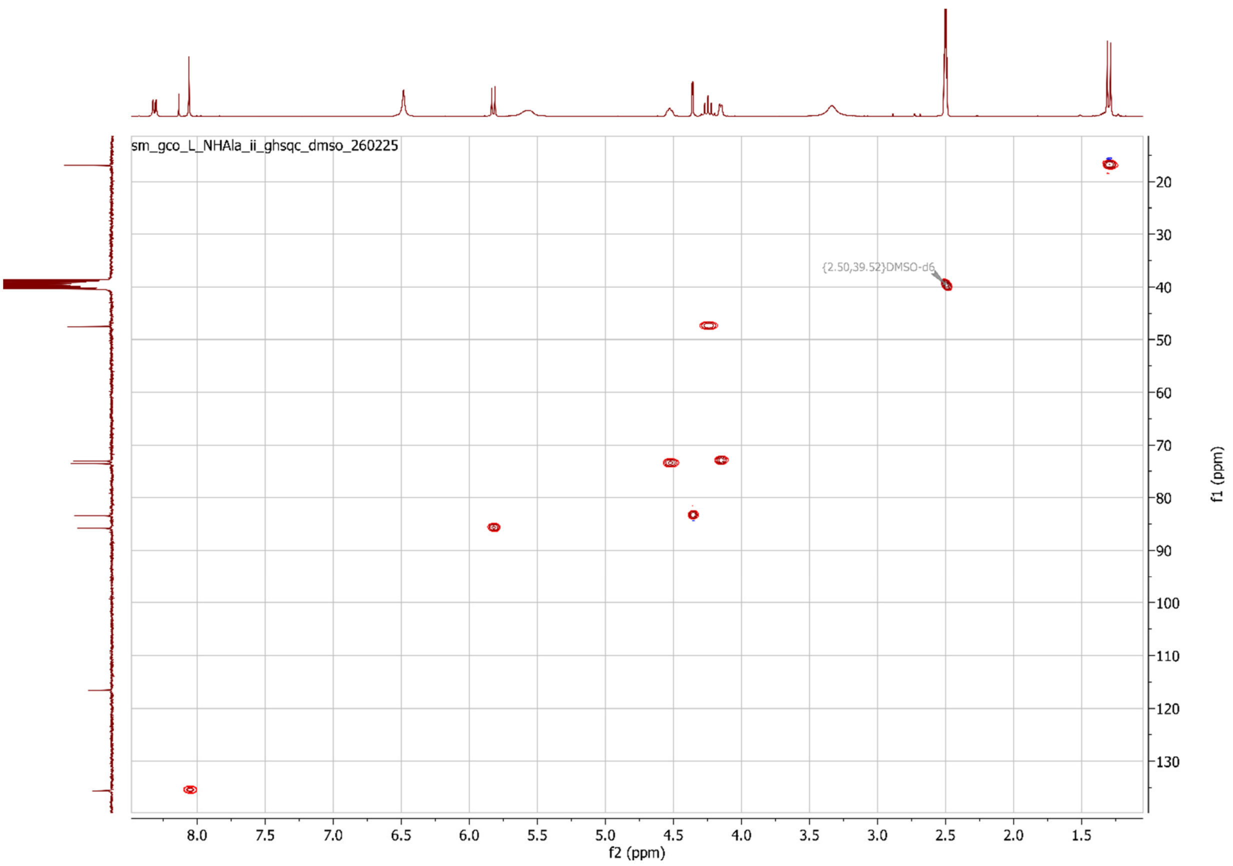

HSQC spectrum of **G-L-Ala** in DMSO-d_6

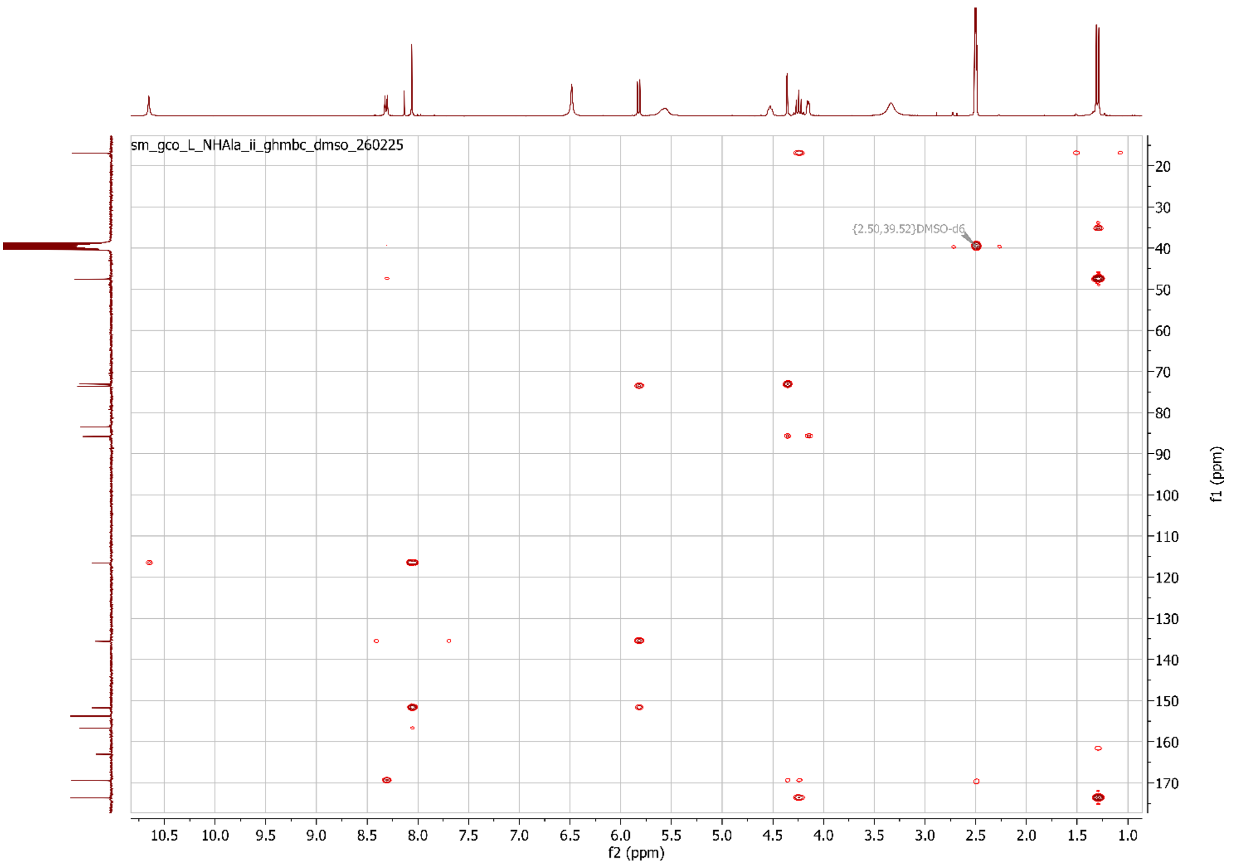

HMBC spectrum of **G-L-Ala** in DMSO-d_6

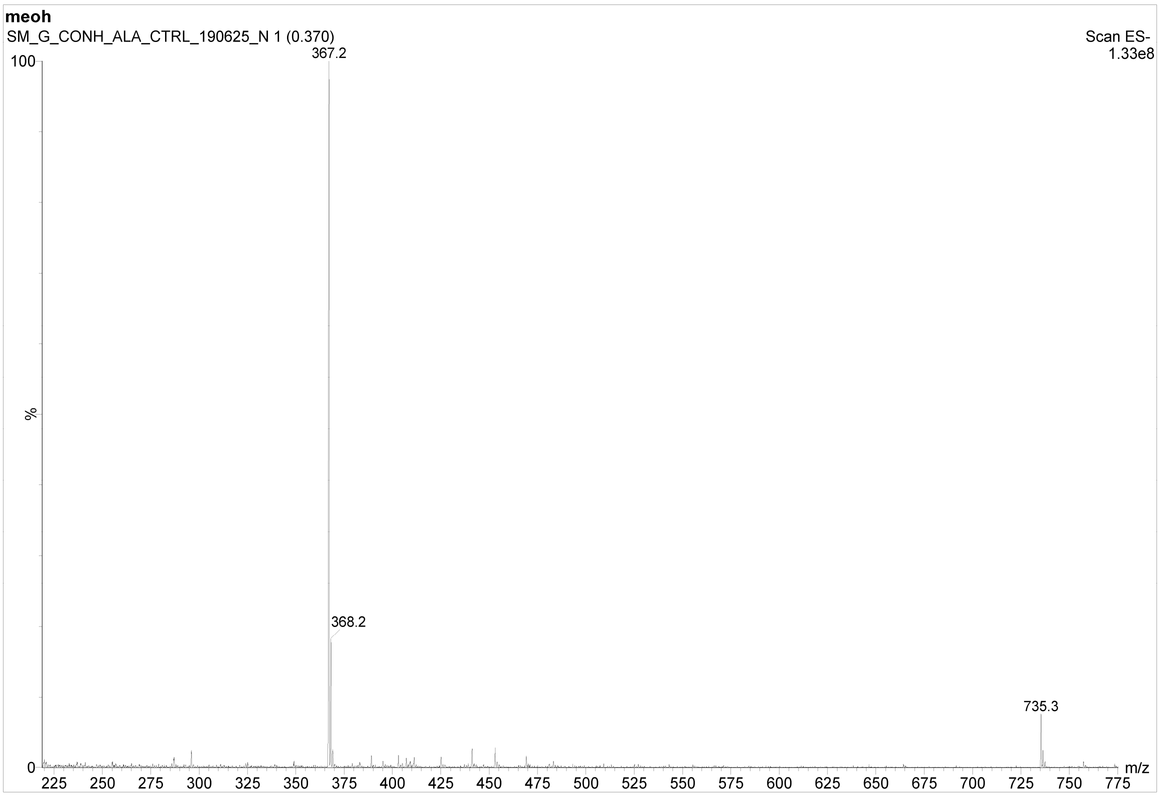

ESI-MS spectrum of **G-L-Ala**

G-D,L-Ala

White solid, 89% yield.

^{1}H NMR (600 MHz, DMSO-d_6) δ 12.50 (bs, 1H, COOH), 10.73 (D) and 10.68 (L) (bs, bs, 1H, guanine NH), 8.31(L) and 8.27 (D) (d, d, J = 7.2 Hz, 1H, -CH-NH-CO-), 8.06 (L) and 8.05 (D) (s, s, 1H, H8), 6.52 and 6.50 (bs, bs, 2H, NH_2), 5.83 (D) and 5.82 (L) (d, d, J = 7.3 Hz, 1H, H1'), 5.59 (bs, 2H, OH), 4.52 (L) and 4.48 (D) (dd, dd, J = 7.3, 4.5 Hz, 1H, H2'), 4.36 (L) and 4.34 (D) (d, d, J = 1.8 Hz, 1H, H4'), 4.28 (D) and 4.24 (L) (p, p, J = 7.3 Hz, 1H, NH-CH-CO), 4.14-4.18 (m, 1H, H3'), 1.30 (L) and 1.29 (D) (d, d, J = 7.3 Hz, 3H, CH_3).

^{13}C NMR (75 MHz, DMSO-d_6) δ 174.35 (D) and 173.75 (L) (COOH), 169.43 (L) and 169.29 (D) (5'CONH), 156.75, 153.83, 151.76 (L) and 151.59 (D) (C4), 135.79 (D) and 135.59 (L) (C8), 116.72 (D) and 116.58 (L) (C5), 85.97 (D) and 85.83 (L) (C1'), 83.53 (D) and 83.47 (L) (C4'), 73.54 (L) (C2'), 73.12-73.07 (D-C2' and D+L C3'), 47.60 (NH-CH-CO), 17.16 (D) and 16.99 (L) (CH_3).

HRMS (MALDI/Q-TOF) m/z: $[M - H]^-$ calcd. for $C_{13}H_{15}N_6O_7$ 367.1002, found 367.1014.

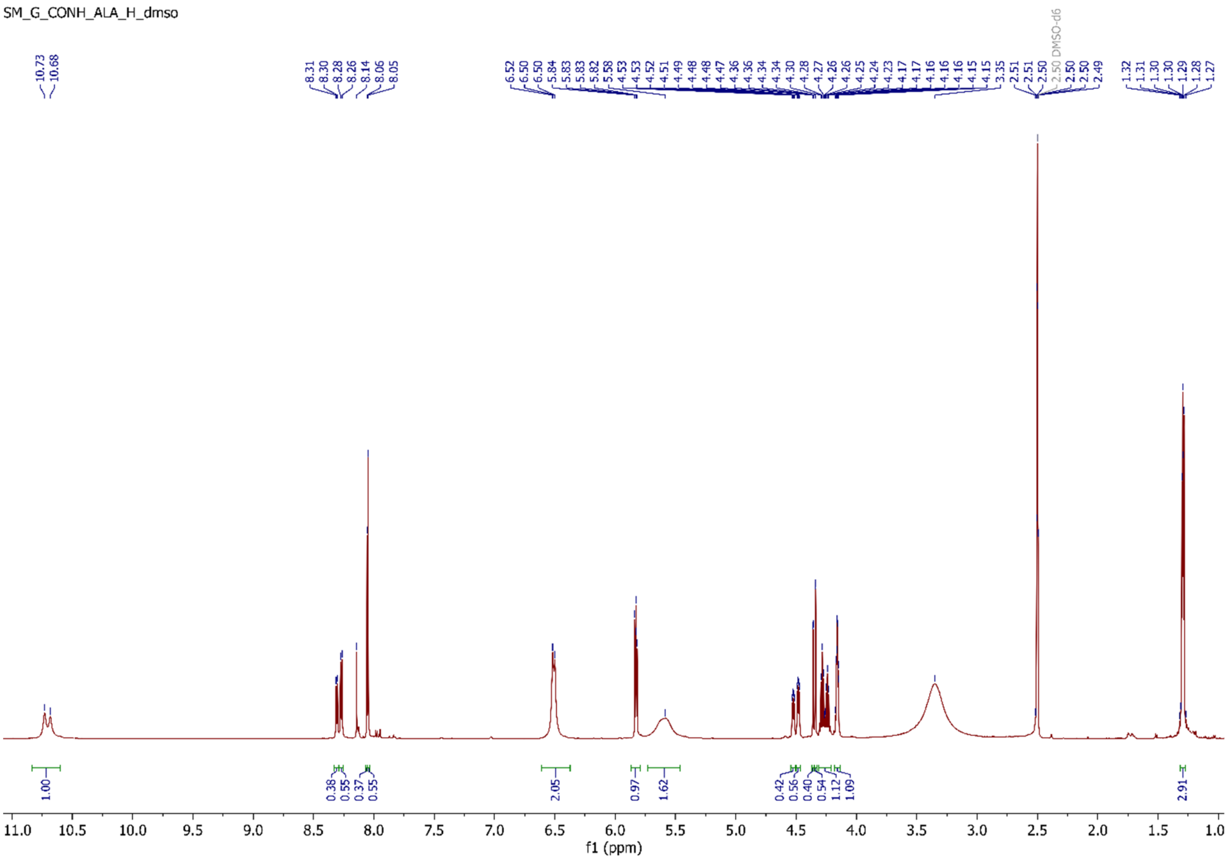

^{1}H-NMR spectrum of **G-D,L-Ala** in DMSO-*d6*

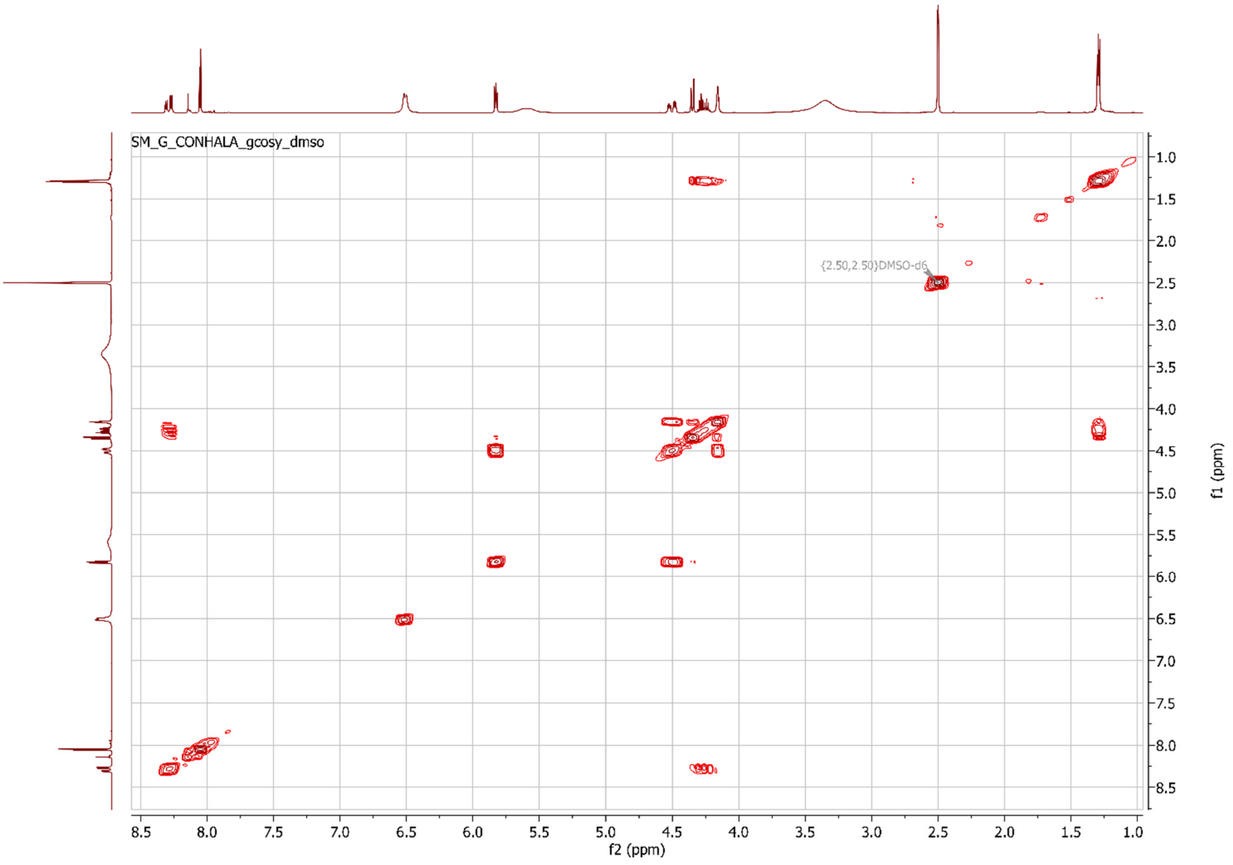

COSY spectrum of **G-D,L-Ala** in DMSO-*d6*

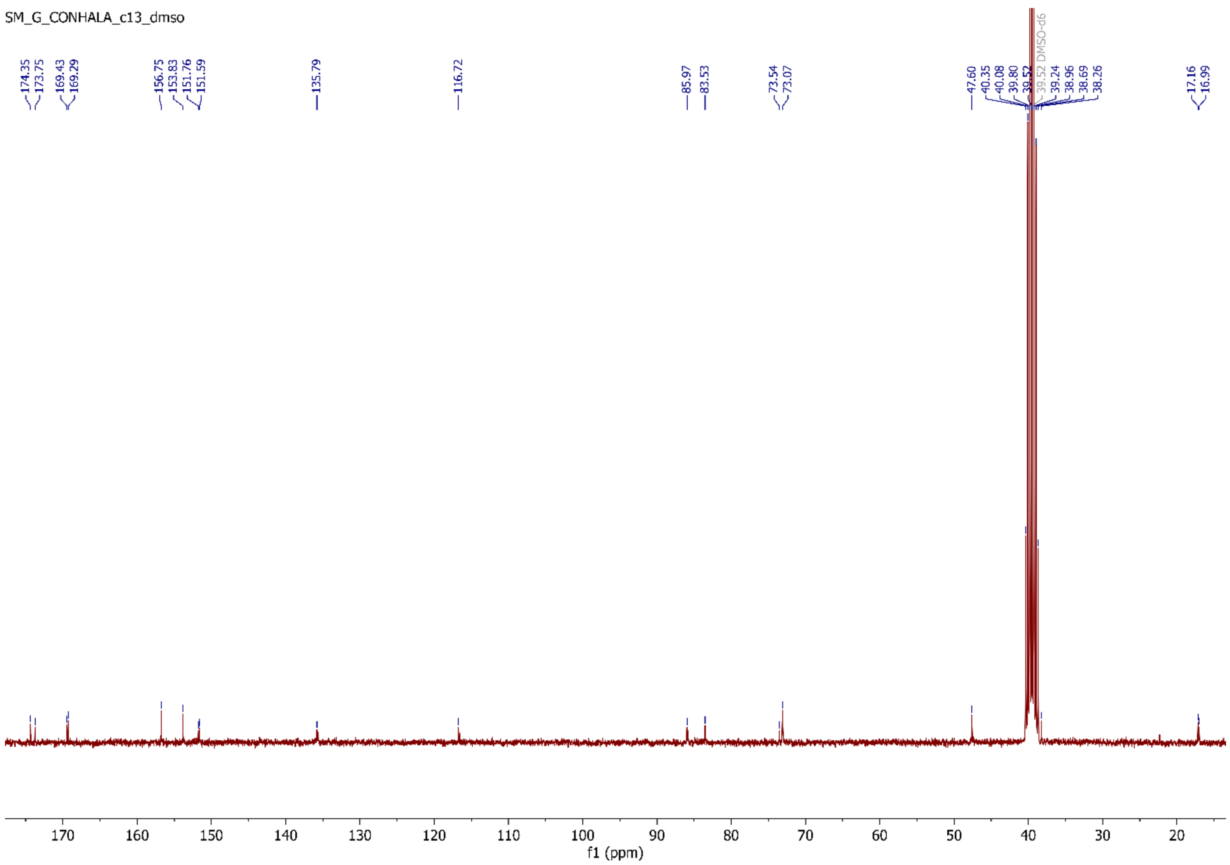

^{13}C-NMR spectrum of **G-D,L-Ala** in DMSO-d_6

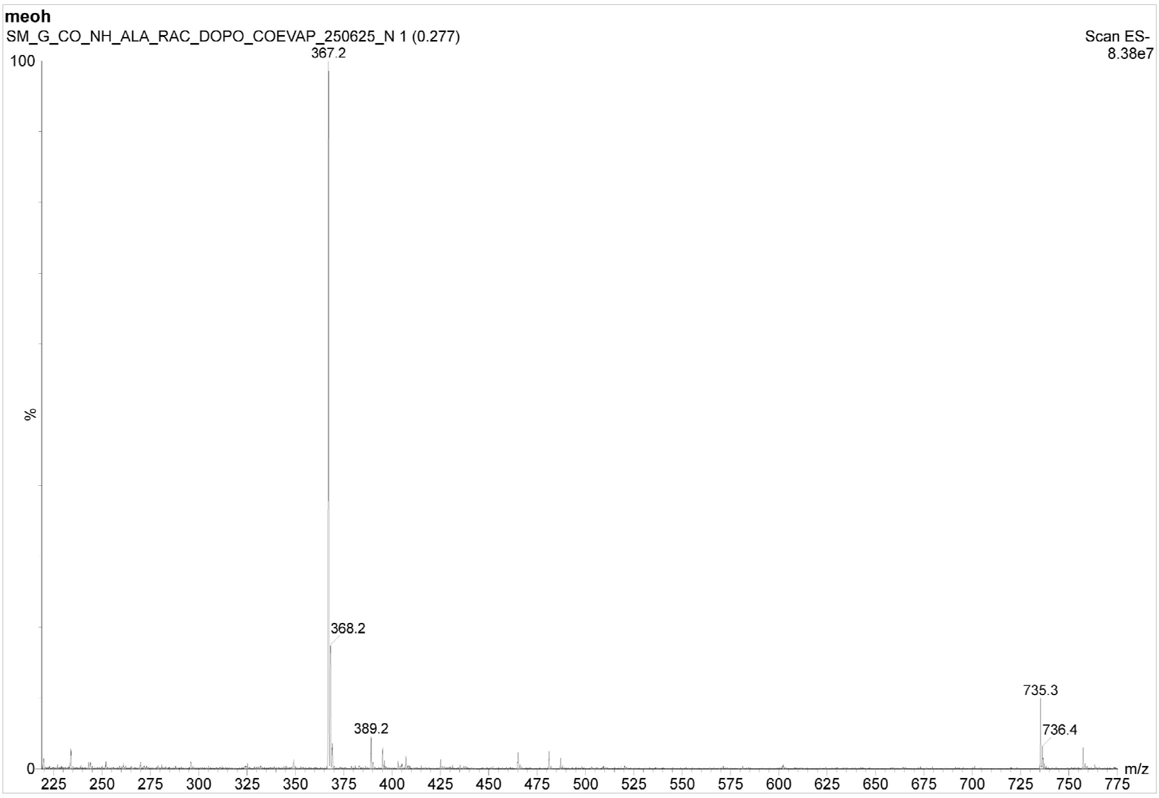

ESI-MS spectrum of **G-D,L-Ala**

2. General procedure for Gel preparation

In a stoppered vial equipped with a magnetic bar were weighted 0.045 mmol of hybrid (15.6 mg or 16.0 mg for **G-Gly** and **G-Ala**, respectively) and suspended in 0.3 mL of KCl (0.15 ÷ 0.35 M). The resulting suspension was sonicated for 2 minutes, 0.75 eq. of KOH (from a 0.4M KOH solution) were added and the mixture was heated to 90°C for 4 minutes under stirring. The resulting solution was left to room temperature until gel formation.

A slightly different procedure was followed for the preparation of gels for rheological analyses. Gels at 1.5% w/V were prepared in glass test tubes: 30 mg of the gelator (**G-Gly**, **G-L-Ala**, **G-D,L-Ala**) were suspended in 1.2 ml of distilled H_2O and 0.64 ml of KCl 1 M were added to obtain a solution 0.354 M, then 0.16 ml of KOH 0.4 M (0.75 equivalents) were added. The mixture, sonicated for 5 minutes, was solubilized by heating to 90 °C for 5 minutes. The solutions were allowed to stand quiescently until gel formation (overnight). The gels were analyzed after 16 h.

3. General procedure for Rheology Analysis

The rheological measurements were performed using an Anton Paar (Graz, Austria) MCR102 rheometer. The gels were directly prepared in the glass test tubes which fit in the rheometer. A vane and cup measuring system was used, setting a gap of 2.5 mm. For the planar plate measurements with gap of 1 mm, the gel was transferred on the plate just before the measurements. Oscillatory amplitude sweep experiments (γ: 0.01–100%) were performed in triplicate at 23 °C using a constant angular frequency of 10 rad/s, 16 h after gel formation. The temperature sweep experiments were set with a temperature range from 23 to 80 °C, using a constant angular frequency of 10 rad/s and γ=1%. After a peak of temperature of 80 °C, the same temperature range was set to cool the sample to 23 °C. The time sweep experiments were recorded after the temperature sweep using a constant angular frequency of 10 rad/s and γ=1%. Frequency sweep tests (ω: 0.1−100%) were performed at 23 °C, setting a constant γ of 0.01% (within the LVE region). The thixotropic behaviour of the hydrogels was assessed with strain-recovery experiments, subjecting the samples to consecutive deformation and recovery steps. The first step (rest conditions) was performed at a constant strain γ = 0.5% (within the LVE region) and at a fixed frequency of ω = 10 rad s^{-1} for a period of 300 s. The deformation step was performed applying a constant strain of γ = 800%, (above the LVE region) for a period of 300 s. The recovery step was performed with the same conditions of the first step for a period of 450 s. Deformation and recovery steps were repeated two times.

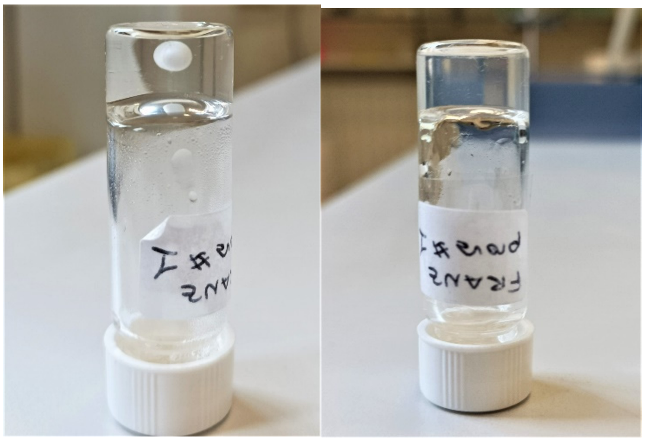

a *b*

Figure S1: ***a***) a freshly prepared **G-Gly** gel 1.3% w/V (the white dot within the gel is the stirring bar introduced into the vial for sample preparation. The bar has then been removed); ***b***) sample ***a*** after 30 days at r.t.

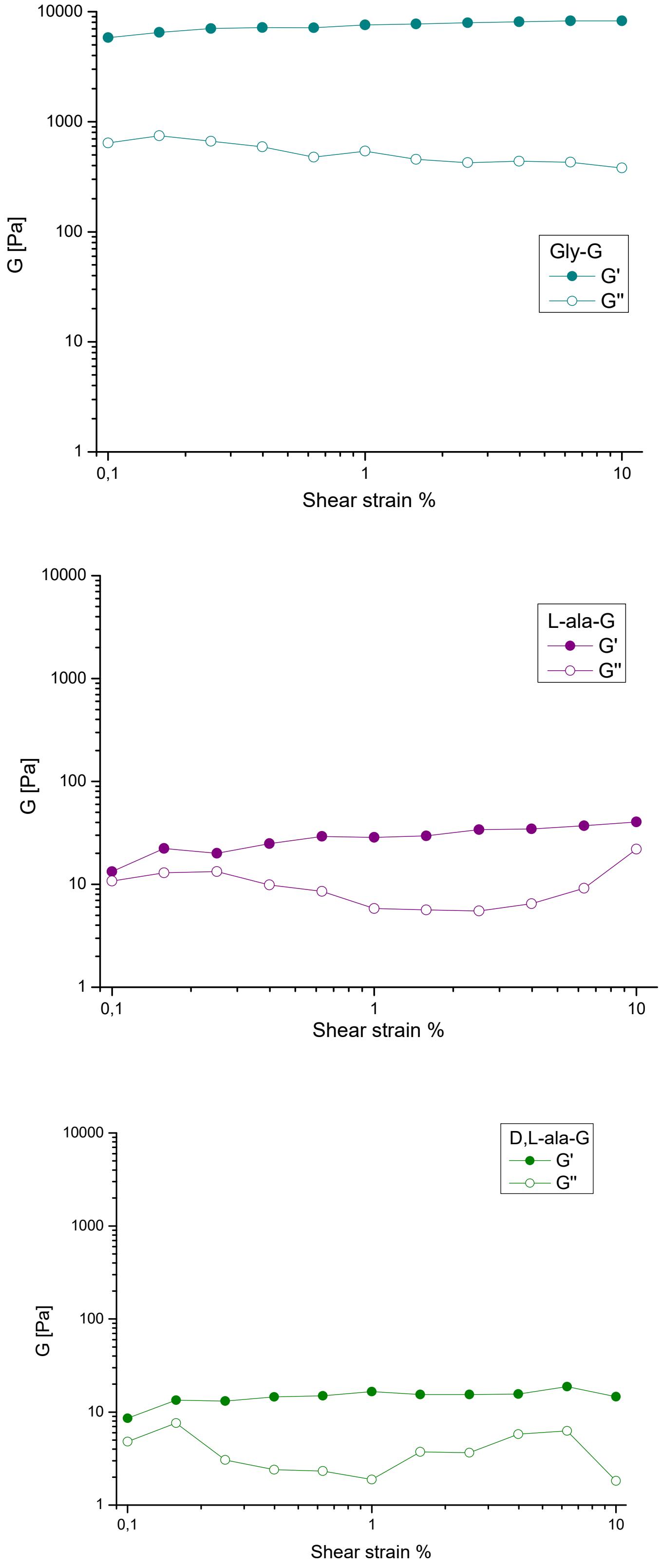

p

Figure S2. Thixotropy sweep test of the hydrogels at the 1.5% w/V. From top to bottom: **G-Gly**, **G-L-Ala**, **G-D,L-Ala**.

4. **Procedures for SEM analysys**

The SEM analyses were acquired with a LEO 1530 FEG (Zeiss, Oberkochen, Germany) using a voltage of 5 kV, an aperture of 30 µm, and a working distance of 9 mm. Prior to analyses, the gels samples (1 mL) were frozen with liquid nitrogen, and freeze-dried. Two procedures were used for the preparation of the aerogels for the SEM analyses. (a) A portion of the aerogels was directly analysed after the freeze-drying process, without any washing. (b) A second portion was washed twice with Milli-Q® water (1-2 mL). For both washes, the solvent was added covering the aerogel and gently swirled; the sample was left at rest for 2 minutes, and the water layer was removed from the top using a pipette.
Then, a portion of the resulting aerogels (before and after the washes) was glued on carbon tape, blown with a nitrogen flow to remove eventual excess of material, and coated with about 20 nm of gold.

5. **General procedures for ECD/UV analysis**

ECD/UV spectra were recorded using a JASCO J-715 spectropolarimeter equipped with a Neslab RTE-111 circulator thermostat (temperature stability ± 0.5°C). Measurements were performed in a 0.001 cm pathlength cell fitted with a thermostated jacket. For loading, the hydrogels were melted and transferred into the cell maintained at 90°C. Spectra were recorded every 10 °C upon cooling from 90 to 10 °C, with an additional point at 5 °C. The temperature was decreased at a rate of 1 °C/min, and a stabilization time of 5 min was allowed at each temperature before measurement. Spectra were recorded from 350 to 190 nm at 100 nm/min and the average of three scans was taken. At 5 °C, the reliability of the CD signal was verified by acquiring spectra after inverting the cell (front-to-back).

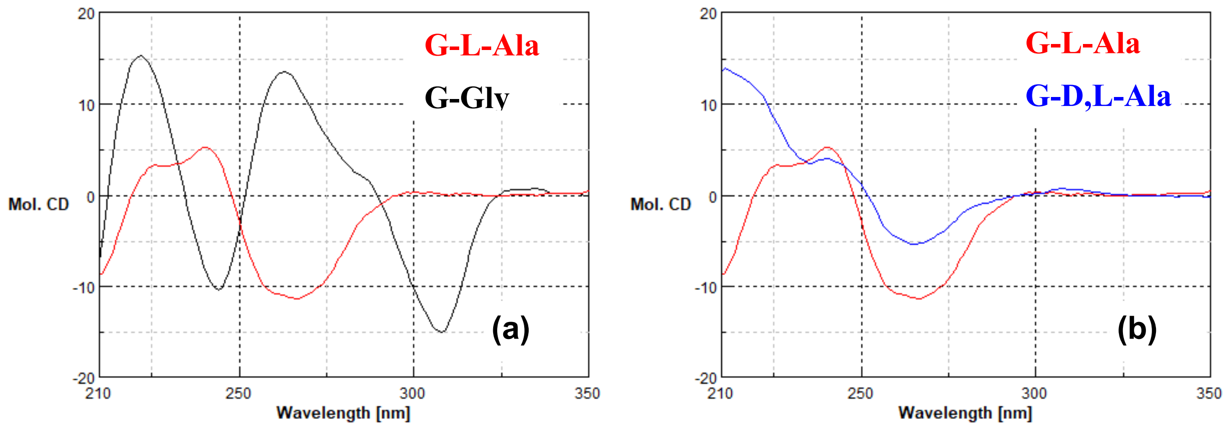

Figure S3. Comparison between ECD spectra of 1.5% w/v hydrogels of **(a) G-L-Ala** (red curve) and **G-Gly** (black curve), **(b) G-L-Ala** (red curve) and **G-D,L -Ala** (blue curve). Spectra were recorded at 20 °C in a 0.001cm cell.

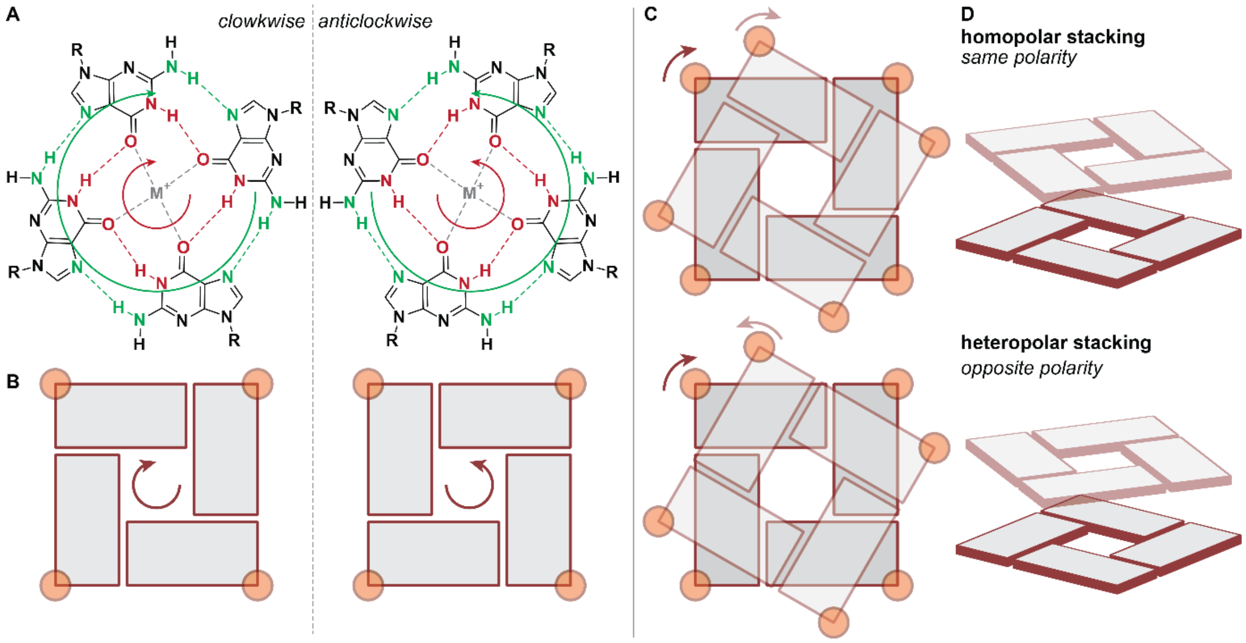

Figure S4. Left. The two enantiotopic faces of a G-quartet are shown in **A**. In **B** a schematic representation is reported. The clockwise and anticlockwise rotation of the hydrogen-bonding pattern according to the donor-to-acceptor direction (*i.e.*, N2-H to N7 and N1-H to O6) are depicted. The two faces are labelled as head (clockwise rotation) and tail (anticlockwise rotation).[48] Right. Top (**C**) and side (**D**) views of two sketched G-quartets stacked in a left-handed helical arrangement with homopolar (head-to-tail) orientation (up), and heteropolar (head-to-head) orientation (down). For the heteropolar stacking the tail-to-tail orientation is not shown.

Figure from E. Largy, V. Gabelica, J.-L. Mergny, *Basics of G-quadruplex structures*, licensed under CC BY-NC-SA 4.0

Source: https://ericlarg4.github.io/Distill_section/docs/guideline.html

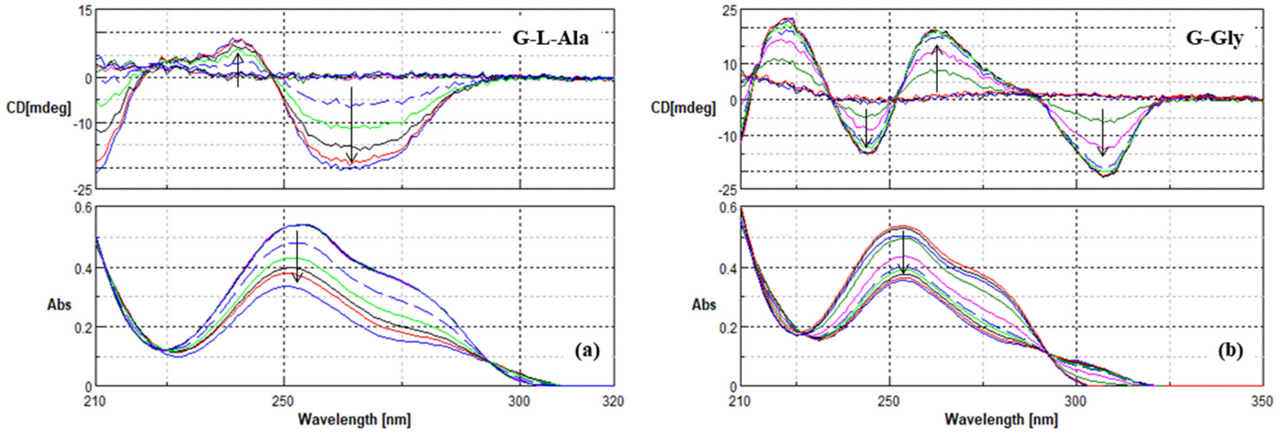

Figure S5. Variable temperature ECD/UV spectra recorded on 1.5% w/v hydrogels of **G-L-Ala (a)** and **G-Gly (b)**. The experiments were performed by lowering temperature from 90 to 5 °C (see experimental part). A 0.001 cm cell was used. The arrows indicate intensity variations of the significant bands upon cooling.

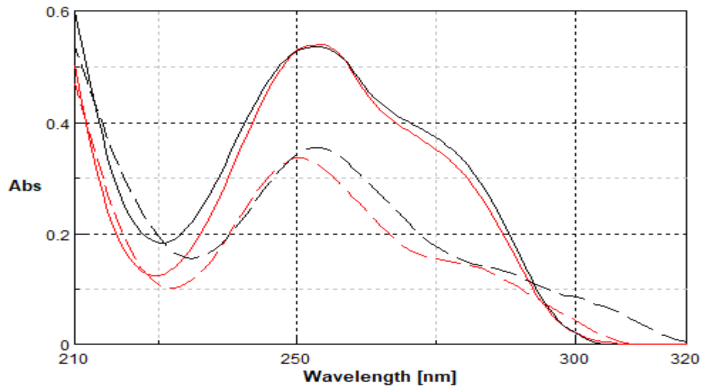

Figure S6. Comparison between UV spectra recorded on 1.5% w/v hydrogels of **G-L-Ala** (in red) and **G-Gly** (in black) at 90 °C (solid lines) and 5 °C (dashed lines). A 0.001 cm cell was used. Spectra were selected from Figure S5.

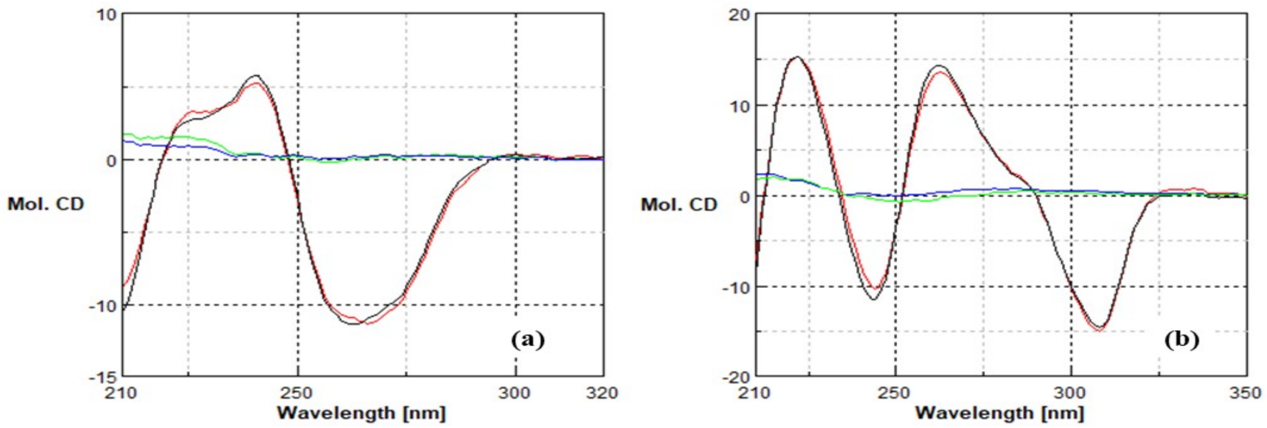

Figure S7. ECD spectra of 1.5% (w/v) hydrogels of **G-L-Ala (a)** and **G-Gly (b)** recorded at different temperatures, showing the reversibility of GQ self-assembly. Blue lines correspond to the initially dissolved samples at 90 °C; red lines were recorded after cooling to 20 °C; green lines were recorded after a second melting at 90 °C; black lines correspond to the second cooling to 20 °C. A 0.001 cm path-length cell was used.
